# Identification and characterization of yellow stripe-like genes in maize suggest their roles in the uptake and transport of zinc and iron

**DOI:** 10.1186/s12870-023-04691-0

**Published:** 2024-01-02

**Authors:** Zizhao Song, Suzhen Li, Yu Li, Xiaojin Zhou, Xiaoqing Liu, Wenzhu Yang, Rumei Chen

**Affiliations:** 1 Crop Functional Genome Research Center, Biotechnology Research Institute, Chinese Academy of Agricultural Sciences, Beijing, 100081 China; 2 College of Food Science and Technology, Hebei Agricultural University, Baoding, 071000 China; 3 CAS Center for Excellence in Biotic Interactions, College of Life Science, University of Chinese Academy of Sciences, Beijing, 100049 China

**Keywords:** Maize, Yellow Stripe-Like transporter, Gene expression, Subcellular localization, Iron, Zinc

## Abstract

**Background:**

Yellow Stripe-Like (YSL) proteins are involved in the uptake and transport of metal ions. They play important roles in maintaining the zinc and iron homeostasis in *Arabidopsis*, rice (*Oryza sativa*), and barley (*Hordeum vulgare*). However, proteins in this family have not been fully identified and comprehensively analyzed in maize (*Zea mays* L.).

**Results:**

In this study, we identified 19 *ZmYSL*s in the maize genome and analyzed their structural features. The results of a phylogenetic analysis showed that ZmYSLs are homologous to YSLs of *Arabidopsis* and rice, and these proteins are divided into four independent branches. Although their exons and introns have structural differences, the motif structure is relatively conserved. Analysis of the *cis*-regulatory elements in the promoters indicated that ZmYSLs might play a role in response to hypoxia and light. The results of RNA sequencing and quantitative real-time PCR analysis revealed that *ZmYSLs* are expressed in various tissues and respond differently to zinc and iron deficiency. The subcellular localization of ZmYSLs in the protoplast of maize mesophyll cells showed that they may function in the membrane system.

**Conclusions:**

This study provided important information for the further functional analysis of ZmYSL, especially in the spatio-temporal expression and adaptation to nutrient deficiency stress. Our findings provided important genes resources for the maize biofortification.

**Supplementary Information:**

The online version contains supplementary material available at 10.1186/s12870-023-04691-0.

## Background

Iron (Fe) and zinc (Zn) are essential for the growth and development of organisms [1]. They act as cofactors and influence photosynthesis, respiration, hormone synthesis, and nitrogen fixation of plants [2–4]. Fe and Zn deficiency decreases cereal production and grain quality, while high levels of Fe and Zn also seriously harm plants. Therefore, Fe and Zn homeostasis needs to be maintained to ensure the growth and development of plants.

The low solubility of Zn and the insoluble form of iron (Fe(III)) prevent plants from absorbing or using these ions passively or directly from the environment. Plants use two strategies to uptake Fe under Fe-deficient conditions [5]. The first strategy (Strategy I) is based on the reduction process, which is used by non-gramineous plants like *Arabidopsis*. The second strategy (Strategy II) is based on the chelation mechanism. Gramineous plants, such as rice and maize, use this strategy to uptake Fe.

Metal ion transporters play a key role in metal ion absorption, intracellular transport, and distribution. Several gene families that are involved in maintaining the homeostasis of Fe and Zn in plants have been identified, including the oligopeptide transporter (OPT) family [6], Zinc-regulated transporters, the Iron-regulated Transporter-like Protein (ZIP) family [7], and the Natural Resistance-Associated Macrophage Protein (Nramp) family [8], among others. OPTs localize to the membrane and different types of substrates. YSLs belong to the OPT protein family, which regulates Fe(III) uptake and the transport of metal-PS complexes in gramineous plants, as well as, the transport of divalent metal ion-NA complexes in non-gramineous plants [9].

The low Fe uptake efficiency observed in yellow-stripe protein 1 (*ys1*) is caused by defects in the Fe^3+^-PS and the Zn-DMA chelate absorption system [10, 11]. *ZmYS1* encodes a membrane protein that mediates Fe(III) uptake [12]. However, the DMA complex is a more suitable substrate for ZmYS1 than the NA complex. Therefore, ZmYS1 is mainly responsible for the uptake of Fe^3+^-DMA. *ZmYS1* is mainly expressed in the epidermal cells of crown and lateral roots and the mesophyll cells of young leaves with severe chlorosis [13]. These findings suggest that ZmYS1 plays a key role in iron uptake and transport. *ZmYSL2* is another YSL gene in maize [14, 15]. It is mainly expressed in the endosperm and encodes a metal-NA transporter that is localized to the plasma membrane. Dysfunction of ZmYSL2 leads to an imbalance of Fe homeostasis and abnormal accumulation of proteins and starch in grains. Therefore, ZmYSL2 plays a key role in the distribution of Fe and kernel development in maize.

The genes in the YSL gene family play a role in transporting divalent metal ion-NA chelates in the circulating distribution and long-distance transport in *Arabidopsis*. It contains eight AtYSLs, among which, AtYSL2 is involved in the lateral transport of metal ions in vascular bundles. [16, 17]. AtYSL1 and AtYSL3 transport Fe through vascular tissues to the above-ground parts [18]. They activate Fe, Zn, and Cu-NA complexes in senescent leaves and redistribute them to reproductive organs, such as flowers and seeds [19, 20]. AtYSL6 and AtYSL4 belong to the same subfamily; the expression of *AtYSL4* in each organ is low and *AtYSL6* is mainly expressed in the above-ground parts, especially in senescent leaves and seeds [21]. The proteins of AtYSL4 and AtYSL6 are involved in Fe homeostasis and distribution, and they prevent metal poisoning in plants [22, 23]. These results indicate that the *AtYSL* genes play different roles in maintaining the homeostasis of metal ions in *Arabidopsis*.

Members of the YSL gene family influence the uptake and distribution of Fe in rice. Out of the 18 rice YSL transporters, researchers have determined the functions of OsYSL2, OsYSL5, OsYSL6, OsYSL9, OsYSL15, OsYSL16, and OsYSL18; they have relatively narrow substrate preferences for metal-PS complexes [24, 25]. For example, OsYSL15 localizes to the plasma membrane and transports Fe^3+^-DMA complexes. It is highly expressed in the epidermis of roots under Fe-deficiency conditions but expressed in the phloem irrespective of the Fe level [22, 26, 27]. OsYSL16 is a membrane protein that is highly homologous to OsYSL15. It absorbs Fe^3+^-DMA from the rhizosphere irrespective of metal levels and distributes Fe^3+^-DMA from the xylem to adjacent cells via vascular bundles [18]. OsYSL9 can transport Fe^2+^-NA and Fe^3+^-DMA. It facilitates the internal transport of Fe during rice seed development, especially for transporting Fe from the endosperm to the embryo [28]. OsYSL2 transports Fe^2+^-NA in the phloem and seeds [29]. OsYSL18 transports Fe^3+^-DMA and is involved in translocating Fe in reproductive organs and phloem in the joints [24]. Like AtYSL4/6, OsYSL6 prevents excessive storage of metals and detoxifies excessive manganese (Mn) [30]. OsYSL5 may also have a detoxification function. These findings indicate that the Fe cycle in rice is as follows: OsYSL15/16 uptakes Fe(III) from the rhizosphere and transports it to the vascular tissue of the root. OsYSL18 translocates Fe from the roots to shoots and transports Fe in reproductive organs. OsYSL9 transports and distributes Fe to leaves. OsYSL2 facilitates the transportation and accumulation of Fe in seeds. OsYSL5/6 compartmentalizes the excess metal for detoxification.

Barley contains five HvYSLs, among which *HvYS1* is expressed in the root epidermis and can transport the Fe^3+^-MA complex; thus, it plays a key role in Fe uptake in the rhizosphere [31, 32]. *HvYSL2* is induced by Fe deficiency in the roots and aboveground parts. It is especially expressed in the endodermis of the roots and might regulate iron-loading in the xylem [33]. HvYSL5 transports Fe^2+^-NA and may be localized in vesicles. It participates in phytosiderophore transport in vesicles [34]. *HvYS1*, *HvYSL2*, and *HvYSL5* are expressed in the roots of barley. However, *HvYS1* is mainly expressed in the root epidermis and only transports Fe^3+^-MA, suggesting that HvYS1 is responsible for the uptake of Fe from the rhizosphere. *HvYSL2* is mainly expressed in the root endodermis and has broad substrate selectivity. It may be involved in Fe transport from the cortex to the pericardium. *HvYSL5* is expressed in all root cells and only transports Fe^2+^-NA. It probably helps maintain Fe homeostasis and prevent excessive Fe uptake by HvYS1.

Although some researchers have investigated the functions of ZmYS1 and ZmYSL2 in maize, a systematic analysis of the members of the ZmYSL family needs to be performed. In this study, we identified the genes of the YSL family in maize. We identified 19 *ZmYSL* genes and analyzed them in detail using bioinformatics. We obtained information on their phylogeny, chromosome location, gene structure, protein motif structure, promoter *cis*-regulatory elements, and gene expression profile. We also examined the tissue expression pattern throughout the life cycle, the level of response to Fe and Zn deficiency, and the subcellular localization of ZmYSLs. Our results provided valuable information that may be used to further study the functions of these genes.

## Results

### Identification and characterization of the ZmYSL family

**Fig. 1:**
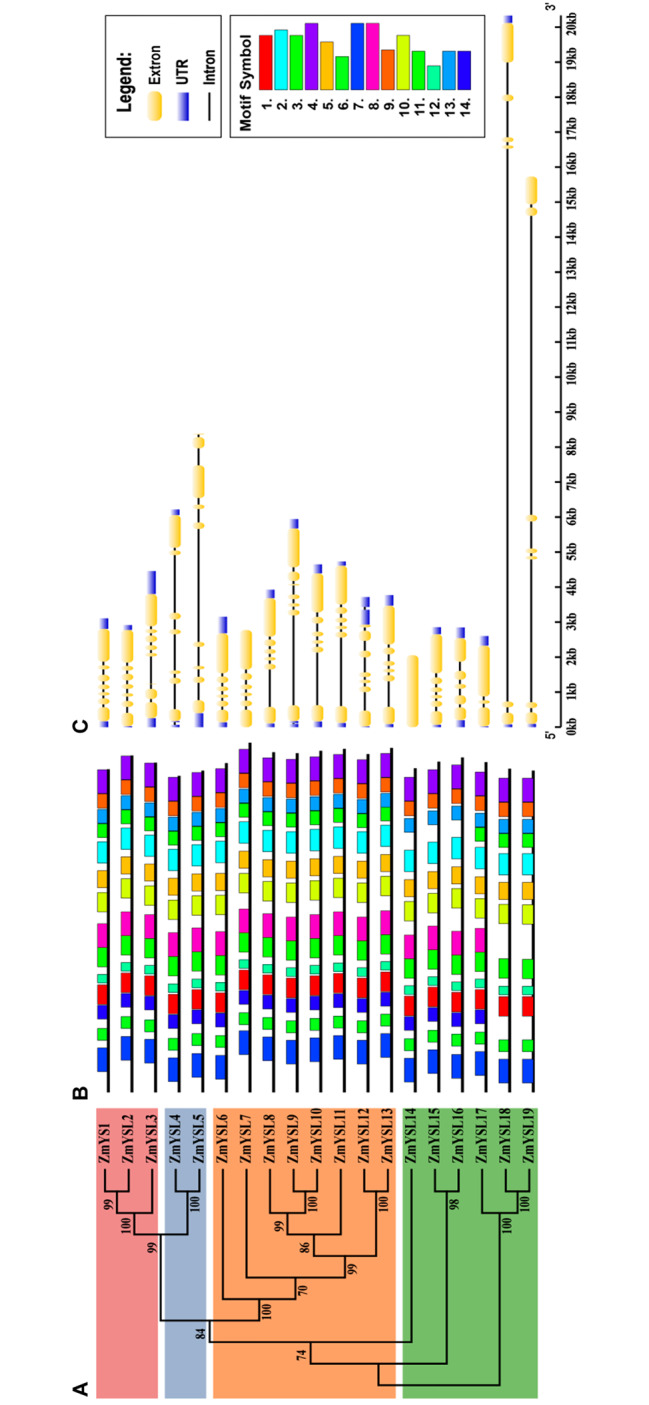
The phylogenetic tree, conserved motifs, and gene structure of the *YSL* gene in maize. (**A**) Phylogenetic tree of the maize YSL protein. The phylogenetic tree of the maize YSL protein was generated using the global alignment program ClustalW. The neighbor-joining method was used for constructing the tree, the Bootstrap method was used for stability testing 1,000 times, and the p-distance was used as the distance model. (**B**) The conserved motif structure of the ZmYSL protein. The size of the box indicates the length of the preserved pattern. The black lines represent non-conservative sequences. (**C**) Gene structure of ZmYSLs. The structures of exons and introns of ZmYSLs were analyzed using the online tool Gene Structure Display Server 2.0. The blue boxes represent the UTR region, the yellow boxes represent exons, and the black lines represent introns

In total, 19 proteins in the ZmYSL superfamily were identified in maize using the Blast program based on the known ZmYS1, AtYSL, and OsYSL as tblastn queries (Table 1). These candidate genes were confirmed by searching the Pfam database for the presence of the OPT subdomain (PF03169.15). The 19 ZmYSL proteins were classified into four subgroups (Fig. 1A). After ZmYS1 and ZmYSL2 were characterized, other ZmYSLs were designated as ZmYSL3 to ZmYSL19, respectively, based on the evolutionary distance between maize and *Arabidopsis* and rice.

**Table 1:** Detailed information of the identified maize YSLs

Gene Name	Gene ID	CDS length	TMs	Protein Characteristic			
				Protein length	*pI*	Mw(Da)	GRAVY
*ZmYS1*	Zm00001d017429	2049	14	682	9.02	74300.97	0.43
*ZmYSL2*	Zm00001d017427	2136	14	711	8.73	76252.53	0.39
*ZmYSL3*	Zm00001d002797	2019	12	672	8.76	75819.69	0.39
*ZmYSL4*	Zm00001d003941	2007	13	668	6.53	72332.64	0.56
*ZmYSL5*	Zm00001d003939	2040	16	679	5.44	72992.28	0.60
*ZmYSL6*	Zm00001d026604	2169	13	695	8.66	75096.76	0.44
*ZmYSL7*	Zm00001d025889	1896	13	738	7.89	79283.15	0.42
*ZmYSL8*	Zm00001d025887	2157	12	718	8.67	77680.64	0.47
*ZmYSL9*	Zm00001d051193	2151	12	716	8.87	77494.52	0.46
*ZmYSL10*	Zm00001d017323	2166	12	721	7.87	78063.07	0.47
*ZmYSL11*	Zm00001d002974	2178	14	725	8.68	78833.19	0.49
*ZmYSL12*	Zm00001d025888	2145	12	714	8.62	77266.48	0.52
*ZmYSL13*	Zm00001d002972	2184	11	727	8.44	79113.19	0.39
*ZmYSL14*	Zm00001d054041	2055	12	684	8.91	75179.89	0.49
*ZmYSL15*	Zm00001d012466	2040	14	698	9.29	75934.57	0.37
*ZmYSL16*	Zm00001d054042	2124	13	707	9.15	76869.03	0.44
*ZmYSL17*	Zm00001d009025	2094	12	697	8.42	74800.18	0.44
*ZmYSL18*	Zm00001d010034	2052	12	683	9.12	74993.01	0.50
*ZmYSL19*	Zm00001d010036	2040	13	679	9.03	74554.05	0.61

TMs, transmembrane domains. Mw, molecular weight. *pI*, isoelectric point. GRAVY, grand average of hydropathicity

The similarity between ZmYSL proteins was found to range from 31.23 to 94.13%, while the length of the ZmYSL proteins was 668–738 amino acid (aa) residues. The molecular weight (MW) of the proteins was 72.3–79.3 kDa, and their grand average hydropathy (GRAVY) values were 0.37–0.61. The isoelectric point (*pI*) values ranged from 5.44 to 9.29, except for the *pI* of the non-functional second subgroup, which exhibited an acidic preference (the *pI* of ZmYSL4 and ZmYSL5 was 6.53 and 5.44, respectively). All other YSL subgroup members were found to be alkaline proteins (Table 1).

### Protein motif structure and genomic distribution of ZmYSLs

All conserved motifs of ZmYSLs (designated as motifs 1–14) were identified, with amino acid lengths ranging from 18 to 50 (Fig. 1B). Additionally, motif predictions were made for the N-terminal before the first TM using default values. We identified a sequence (SVERAFADQPVPPWREQLTVR), which was 21 amino acids long, similar to the YSL feature sequence K (F/R) L (T/P/A) (Y/F) PSG (T/L/S) ATA (V/M/H) LIN.

The gene structure of *ZmYSL* was analyzed by determining the arrangement of exons and introns (Fig. 1C). The length of the open reading frame was 1,896–2,184 base pairs, and the number of exons varied from 1 to 9. *ZmYSL5* had the largest number of introns and exons, *ZmYSL14* had no introns, and *ZmYSL18* and *ZmYSL19* had very long introns.

**Fig. 2:**
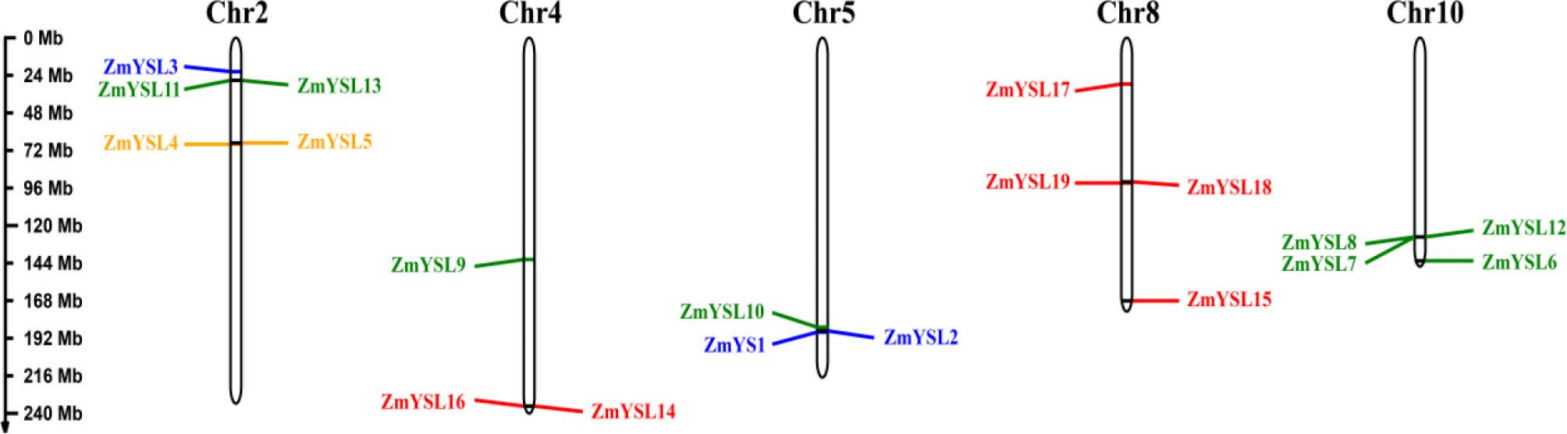
Chromosome distribution of the *YSL* gene family in maize. The location of 19 *YSL* genes on the chromosome was mapped according to the maize genome. The chromosome numbers are shown at the top of each chromosome, and the scale is in megabases (Mb). Duplicated *YSL* gene pairs are linked by a brown line between the genes. Blue indicates the first subgroup gene, orange indicates the second subgroup gene, green indicates the third subgroup gene, and red indicates the fourth subgroup gene

All 19 identified *ZmYSL* genes were almost evenly distributed on chromosomes 2, 4, 5, 8, and 10, according to its genomic mapping information (Fig. 2). The non-functional second subgroup proteins were found only on chromosome 2, and the fourth subgroup genes were found only on chromosome 8.

### Phylogenetic analysis of YSL members

**Fig. 3:**
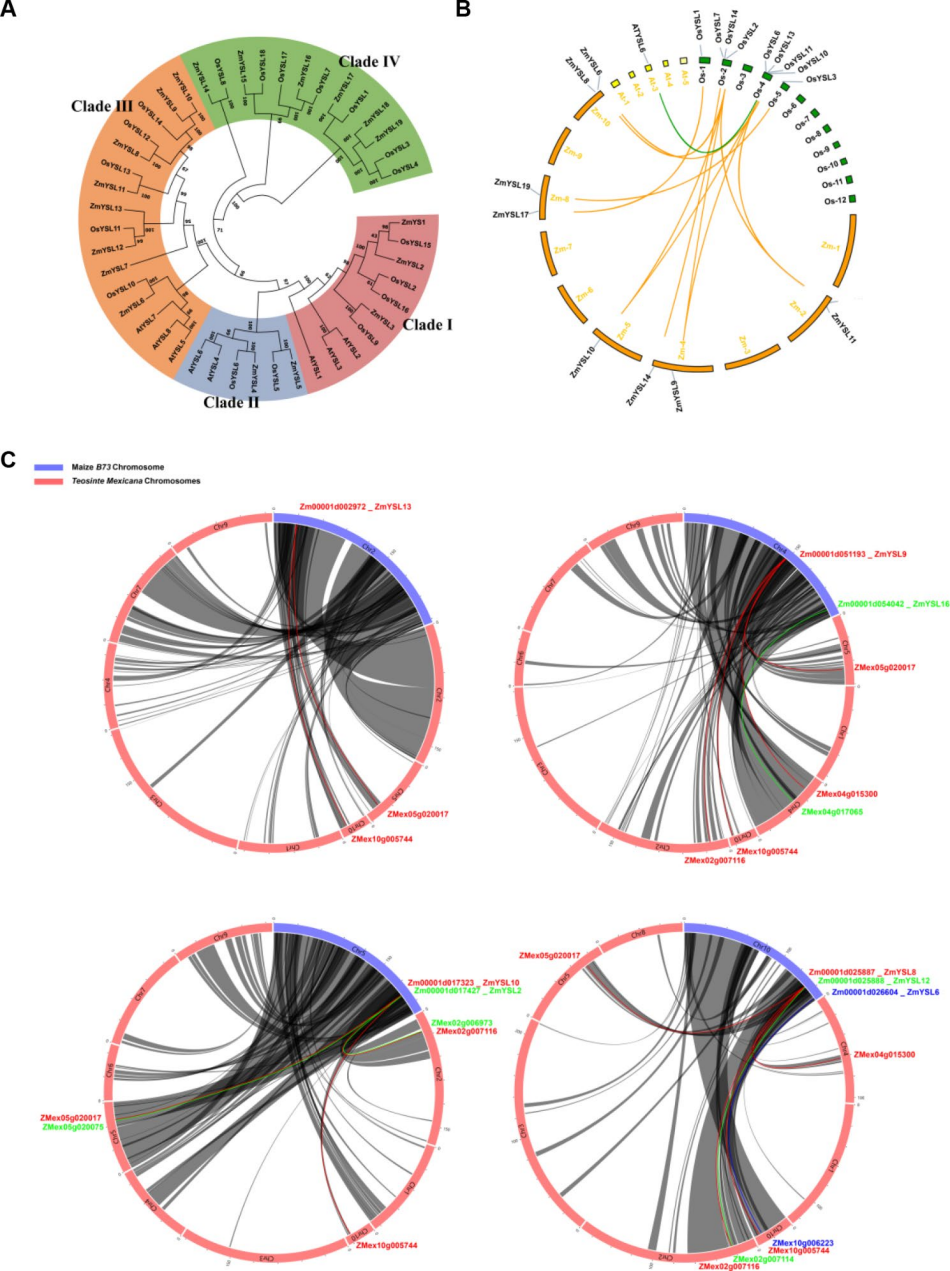
Phylogenetic relationship and collinearity analysis were conducted between the YSL protein of *Zea mays* and the YSL proteins of *Arabidopsis thaliana* and *Oryza sativa*. (**A**) A phylogenetic analysis of YSL proteins in maize (ZmYSL), *Arabidopsis* (AtYSL), and rice (OsYSL) was performed using MEGA10.0 and the neighbor-joining (NJ) method. A bootstrap test of 1,000 replicates was used for internal branch reliability; the scale bar represents the difference in site ratio (p-distance). (**B**) A collinearity analysis was performed on the YSL family in maize, rice, and *Arabidopsis*. The chromosomes of maize, rice, and *Arabidopsis* are marked by orange, green, and yellow boxes, respectively. The collinear relationship of rice with maize and *Arabidopsis* is indicated by orange and green lines, respectively. (**C**) Collinearity analysis of YSL family in maize B73 and Teosinte. The chromosomes of maize B73 and Teosinte are shown in purple and pink boxes, respectively. The collinearity and corresponding gene ID are shown in the same color

The protein sequences of 19 ZmYSLs, eight AtYSLs, and 18 OsYSLs were used to construct a phylogenetic tree (File S1, Fig. 3A). The results showed that ZmYSLs and OsYSLs were distributed across four different branches (branches I-IV), and AtYSLs was distributed among the first three branches. Among the maize branches, the third branch was the largest (containing eight genes), and the second branch was the smallest (containing two genes). The largest branch had an extensive chromosome distribution, while the smallest branch, which contains two non-functional genes, had a narrow and closely located chromosome distribution.

A collinear relationship among maize, *Arabidopsis*, and rice was established based on phylogeny (Fig. 3B). The *YSL* genes of *Arabidopsis* were distributed on chromosomes 1, 3, 4, and 5, the *YSL* genes of rice were distributed on chromosomes 1, 2, 4, 5, and 8, and the *YSL* genes of maize were distributed on chromosomes 2, 4, 5, 8, and 10. The 19 ZmYSLs did not exhibit collinearity with AtYSLs, but seven ZmYSLs showed collinearity with OsYSLs; we also found collinearity between AtYSL6 and OsYSL6. The function of the collinear YSL in maize and rice might be similar, and the function of the collinear gene in maize may be predicted by the known function of YSL in rice. This might be because maize and rice are both grasses and use Strategy II for the uptake of Fe. In contrast, *Arabidopsis* uses Strategy I for the uptake of Fe. Thus, a greater genetic evolutionary distance might occur between *Arabidopsis* and maize. To obtain comprehensive evolutionary information on the widely cultivated maize cultivar B73 and its ancestor teosinte, given the constraints of a small assembly genome size and lack of annotation file for teosinte, we conducted a collinearity relationship between B73 and Teosinte (*Zea mays* ssp. *Mexicana*) using the recently published ZEAMAP database (Fig. 3C). Only eight B73 genes which are distributed across Chromosomes 2, 4, 5 and 10 exhibit synteny with nine *Mexicana* genes. ZmYSL6, ZmYSL12, and ZmYSL16 have identical genes in *Mexicana*. The two teosinte genes eventually merged into a single gene, ZmYSL2. Four genes (*ZmYSL8*, *ZmYSL9*, *ZmYSL10*, and *ZmYSL13*) have complex collinearity with ZMex02g007116, ZMex04g015300, ZMex05g020017, and ZMex10g005744 in *Mexicana*. These four genes in *Mexicana* may be scrambled and recombined into four new genes (*ZmYSL8*, *ZmYSL9*, *ZmYSL10*, and *ZmYSL13*) in B73. *ZmYSL2*, *ZmYSL6*, *ZmYSL12* and *ZmYSL16* are highly conserved in evolution, suggesting that these four genes play a crucial role in zinc/iron uptake and transport, and they may be the candidate genes for efficient breeding of zinc/iron.

### Transmembrane domain analysis

**Fig. 4:**
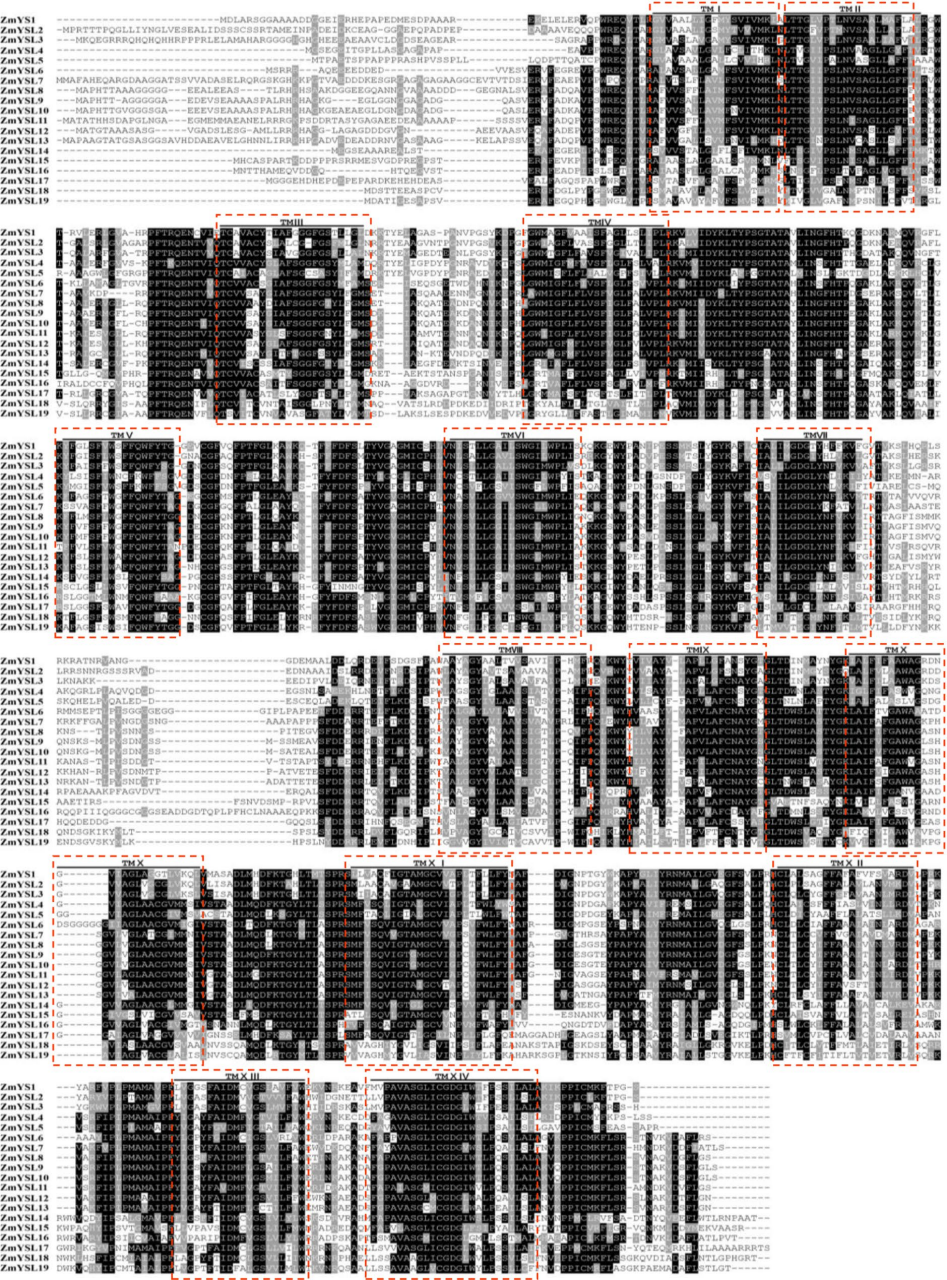
The amino acid alignment and predicted transmembrane domains of the ZmYSL proteins. The 19 ZmYSL protein sequences were aligned using the multiple sequence alignment method. Conserved amino acid residues were highlighted using Boxshade. The 14 summarized transmembrane domains are indicated by a red dotted box and accompanied by ordinal numbers above

The number of transmembrane domains in different ZmYSL proteins ranged from 11 to 16. The number of summarized transmembrane domains was 14, and the amino acids in these domains were highly conserved (Fig. 4). Two highly variable regions appeared between the N-terminal and the seventh and eighth transmembrane domains. These two regions may be related to the substrate selectivity of ZmYSL transporters. ZmYSL4, ZmYSL13, ZmYSL16, and ZmYSL19 have an extracellular N-terminal and an intracellular C-terminal; ZmYSL5 and ZmYSL17 have extracellular N- and C-terminals; ZmYSL6 and ZmYSL7 have an intracellular N-terminal and an extracellular C-terminal. The rest of the ZmYSLs have intracellular N- and C-terminals.

### Analysis of the promoter *cis*-regulatory elements

**Fig. 5:**
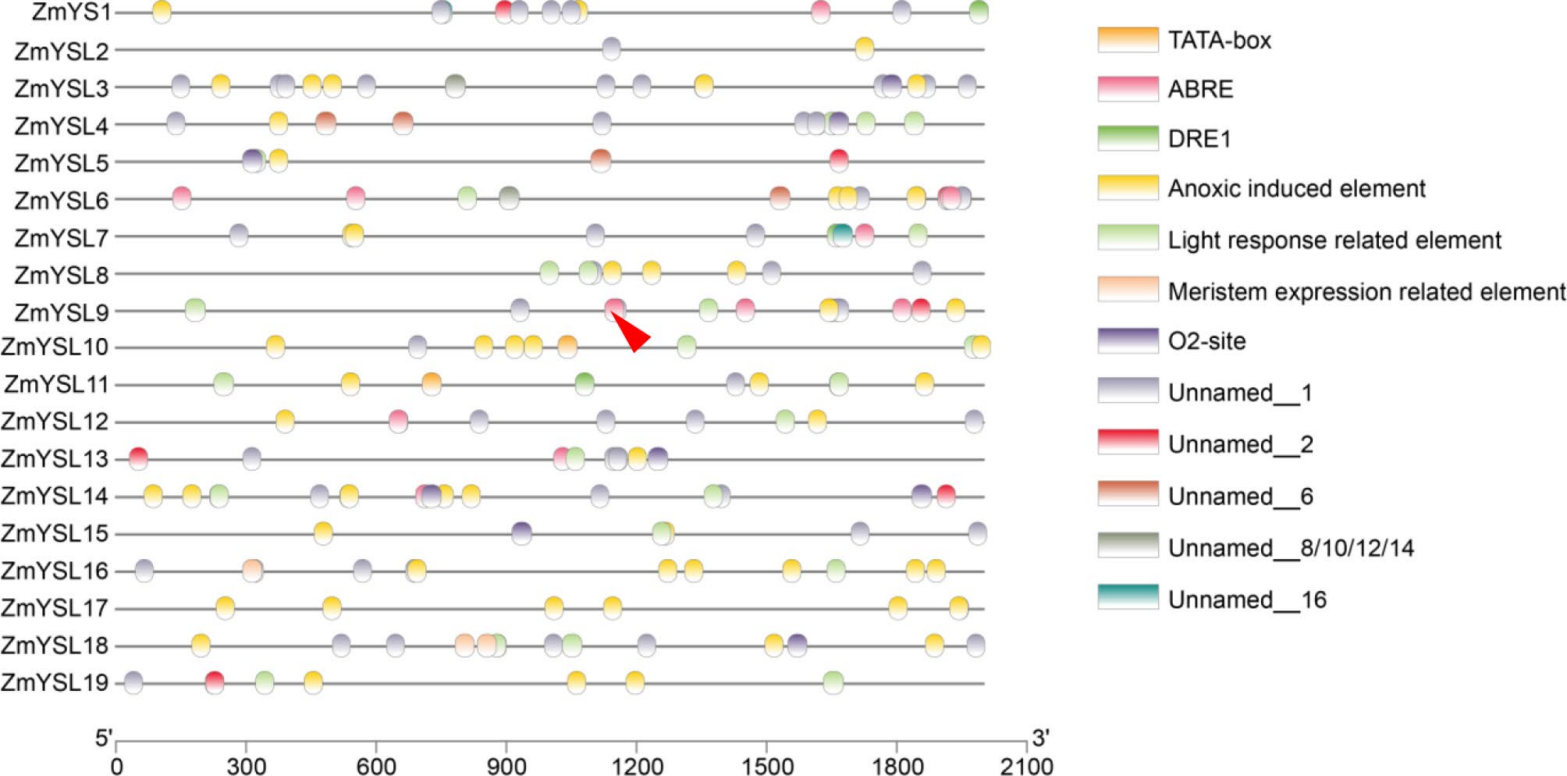
Predictive analysis of *cis*-regulatory elements of the *ZmYSL* promoter. The black line represents the promoter length, and the circles of different colors represent different *cis*-regulatory elements. The red triangle indicates the ABRE element in the promoter of ZmYSL9 that has the same binding motif as IRO2

*Cis*-regulatory elements (CREs) are important elements in gene expression and play a role in regulating plant growth and development as well as stress response. To analyze the *cis*-regulatory elements, the 2000 bp sequences upstream of the ATG of the 19 ZmYSLs were used as promoters and submitted to PlantCARE for prediction (Fig. 5, Table S1) and submitted to MEME-AME to analysis the enrichment motif on the promoters (Table S2). Elements got from PlantCARE are totally classified into five categories (DRE into ABA responsiveness element and GC-motif into Anoxic induced), Forty-nine motifs were enriched by MEME-AME program. Twenty-one motifs (about 43% of forty-nine motifs) are GC-rich motifs and many of them related to ERF transcriptional factor. In PlantCARE, the GC-motif was also detected with a consensus of CCCCCG and annotated as “Enhancer-like element involved in anoxic specific inducibility”.

Ethylene signaling response involved in resistance to hypoxic stress due to the mechanism by which ethylene signaling controls rapid oxygen responses in plants. These results and other ARE indicate that YSL is involved in plant respiration due to its ability to transport zinc and iron [35]. Additionally, the presence of multiple dehydration response elements (DREs) and abscisic acid response elements (ABREs) suggests that some YSLs may be involved in the regulatory pathway of ABA hormone (Fig. 5). Exogenous ABA can systematically activate Fe stored in root epidermis and vacuoles and induce the expression of long-distance Fe transport genes to promote effective transport of Fe to the shoot [36, 37]. Recent studies have shown that bZIP19 regulates ZmABI29 through the ABRE element, which finally affect maize grain development in ABA hormone pathway [38] Also, the elements are related to light response, which may be relevant to the transport of Fe and oxygen implicated in photosynthesis [38]. Four motifs with a lot of A annotated as REM19 may related to reproductive meristem function, and some meristem expression related elements were also obtained through PlantCARE. Six motifs with a consensus of GAGA or CTCT annotated as BPC may related to function of hormonal signaling, stress, circadian oscillations, and sex determination processes. Eight motifs with a consensus of CTTT may involve in Dof regulatory network and function in seed development, tissue differentiation and metabolic regulation. The Fe-related transcription factor (IRO2) is a positive regulator of Fe uptake when Fe deficiency occurs [39, 40]. During promoter analysis, we identified a sequence (CACGTGG) that can bind to IRO2 in ZmYSL9 (Fig. 5). Our results indicated that ZmYSL9 might participate in regulating Fe deficiency. These findings suggested that ZmYSLs are extensively involved in the growth and development of maize throughout its life cycle.

### The expression profile of *ZmYSL* in maize

**Fig. 6:**
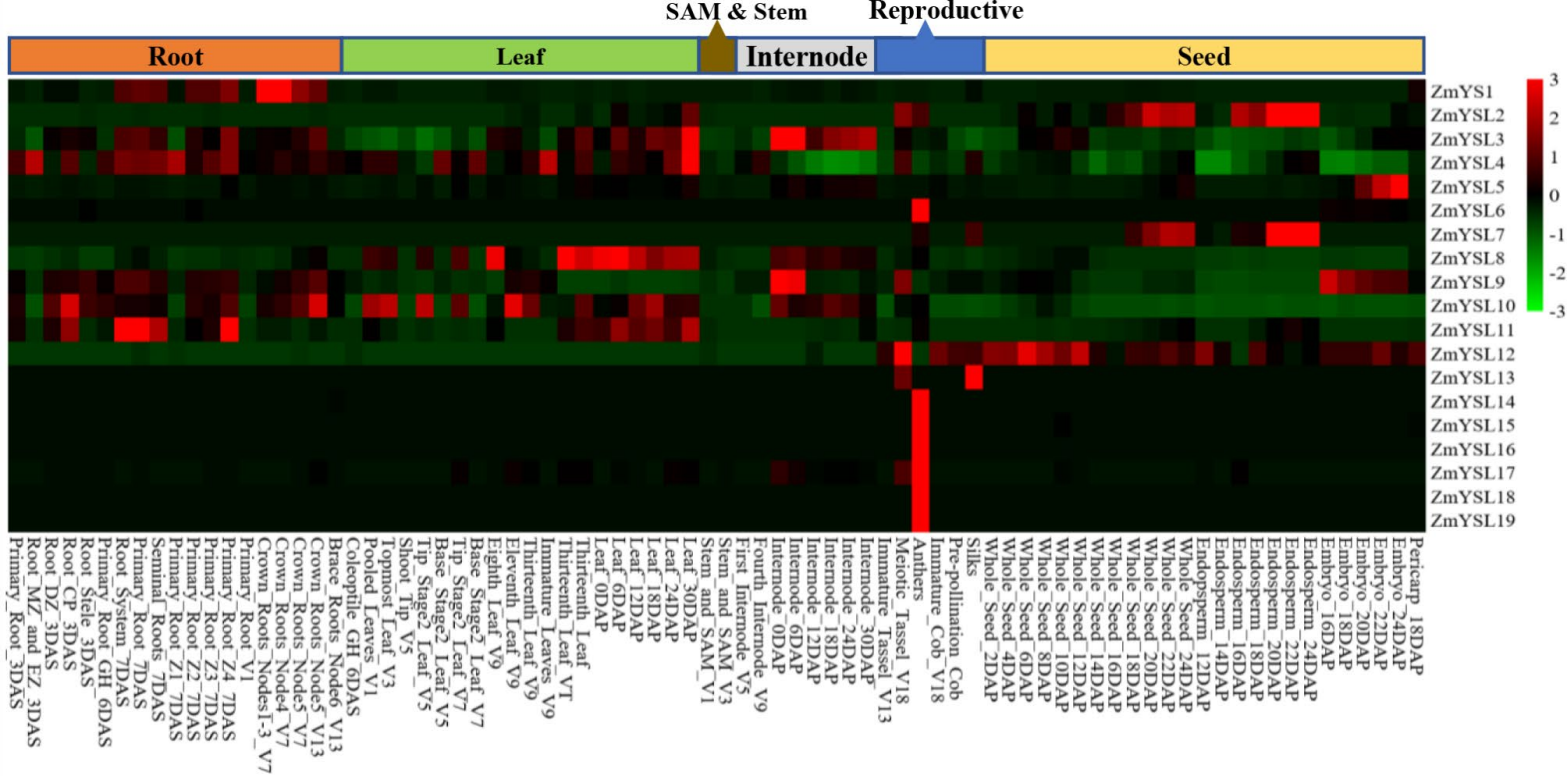
A heatmap of the expression of the *ZmYSL* genes in different tissues and developmental stages. The heatmap was generated from RNA-Seq data of 80 samples collected from the roots, leaves, SAMs & stems, reproductive organs, and seeds at different developmental stages of maize. The FPKM values were normalized at the gene level. The color bar to the right of the heat map shows the fold change, where green represents downregulation, black represents no change, and red represents upregulation

**Fig. 7:**
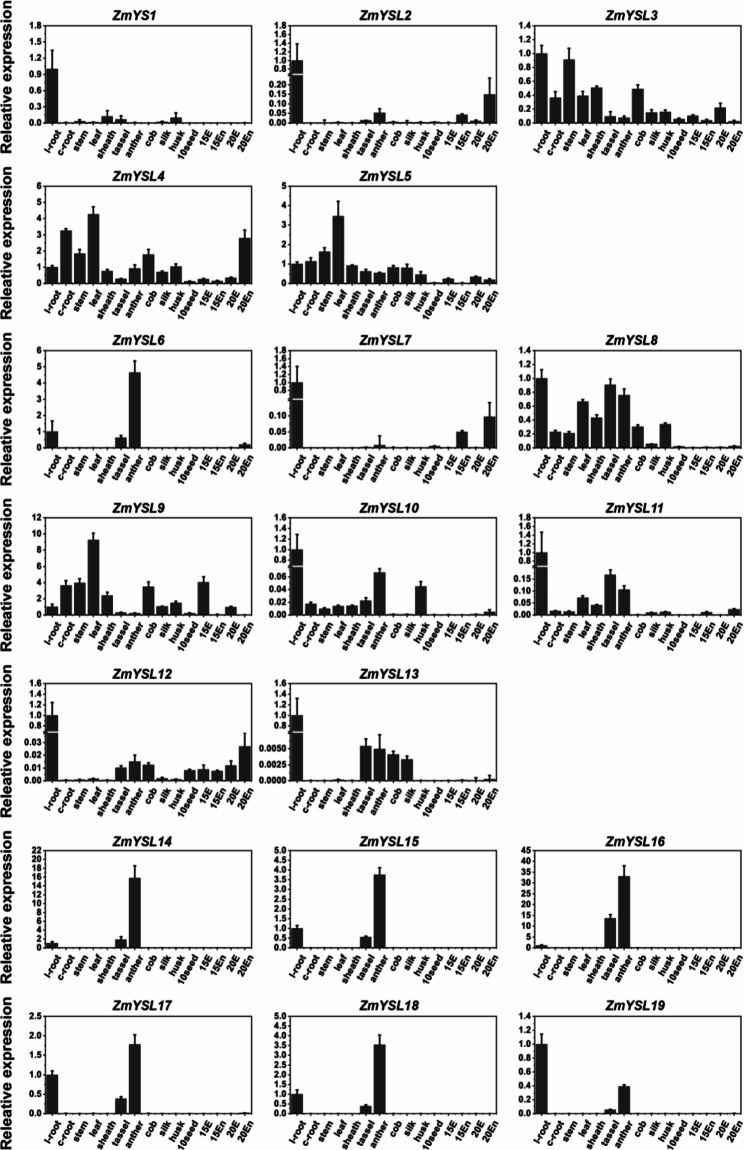
Spatiotemporal expression profiles of the *ZmYSL* genes. Real-time quantitative PCR was performed to analyze the expression of the YSL gene in 15 tissues of maize. Total RNA was extracted from the endosperm (En) and the embryo (Em) at the indicated days after pollination (15 and 20 DAP), as well as, from different organs, including l-root (lateral root), c-root (crown root), stem, leaf, sheath, tassel, anther, cob, silk, husk, and seed (10 days after pollination). The *Actin1* gene in maize was used for normalizing the relative expression of each gene. The error bars indicate the standard deviation

The expression of 19 *ZmYSLs* was determined in 80 different tissues and developmental stages using public RNA-Seq data (Fig. 6). The results showed that *ZmYS1* was mainly expressed in the roots of maize, and its expression increased in the primary roots. *ZmYS1* was also expressed in the crown roots at the seven-leaf stage and in the first three nodes, indicating that ZmYS1 plays an important role in morphogenesis during vegetative growth. *ZmYSL2* was predominantly expressed in the endosperm (Fig. 6) [14, 15]. In our study, the expression pattern of *ZmYSL7* was very similar to that of *ZmYSL2* (Fig. 7), suggesting that they might have the same function.

*ZmYSL3* and *ZmYSL11* were expressed in roots and shoots (Fig. 6), suggesting they might be involved in iron and zinc long-distance transportation through the vascular bundles, and the transportation and distribution of iron and zinc between old and new leaves. *ZmYSL4* was predominantly expressed in roots and leaves (Fig. 6), and belonged to branch II, suggesting that it might be involved in detoxification in plants. *ZmYSL5* and *ZmYSL9* were also highly expressed in embryos, with their expression periods complementing each other (Fig. 6), which might be related to seed germination. *ZmYSL9* was also highly expressed in the nodes, where it might facilitate the short-term storage of iron after transportation through vascular tissue. The expression of *ZmYSL8* was higher in the new leaves before pollination, whereas *ZmYSL10* was predominantly expressed in leaves after pollination (Fig. 6). The expression levels of *ZmYSL4*, *ZmYSL10*, and *ZmYSL11* were higher in the radicle three days after sowing (Fig. 6), which suggested a supplementary function of *ZmYS1*. *ZmYSL6* and *ZmYSL14*-*ZmYSL19* were highly expressed in anthers, while *ZmYSL13* was highly expressed in silk. *ZmYSL2*, *ZmYSL13*, and *ZmYSL17* were also expressed in tassels (Fig. 6). These genes, which are specifically expressed in silk, tassel, and anther, might be related to the reproductive activity of maize.

To better understand their tissue-specific expression, we quantified the gene expression in 15 different tissues of maize via qPCR (Fig. 7). The qPCR results differed slightly from the expression profile mentioned above, and thus, the qPCR assays provided additional information to the RNA-sequencing results. In our study, five *ZmYSL* genes, including *ZmYSL3*, *ZmYSL4*, *ZmYSL5*, *ZmYSL8*, and *ZmYSL9*, were expressed in all tissues (Fig. 7), especially in the tissues of leaves. Six genes, including *ZmYSL6* and *ZmYSL14*-*ZmYSL18*, were predominantly expressed in the tassel and anther, and *ZmYSL19* was also expressed in these two tissues (Fig. 7). Finally, eight genes, including *ZmYS1*, *ZmYSL2*, *ZmYSL7*, *ZmYSL10*-*ZmYSL13*, and *ZmYSL19*, were predominantly expressed in the roots (Fig. 7). These expression patterns suggested that the *ZmYSL* genes play specific roles during the growth and development of maize. Genes that are primarily expressed in reproductive organs, such as the tassel and anther, are probably associated with the reproductive functions of maize; among them, *ZmYSL14* and *ZmYSL16* might play a more pivotal role. Specifically, the expression of *ZmYSL14* and *ZmYSL16* in the anther was extremely high; their expression was 18 and 40 times higher than that in the root, respectively (Fig. 7). The level of expression of *ZmYSL2*, *ZmYSL5*, *ZmYSL7*, *ZmYSL9*, and *ZmYSL12* in the embryo or endosperm was relatively higher than the expression of other *ZmYSL* genes (Fig. 7), which might be related to the development and germination of maize seeds.

### The response of *ZmYSLs* to the deficiency of Fe and Zn

**Fig. 8:**
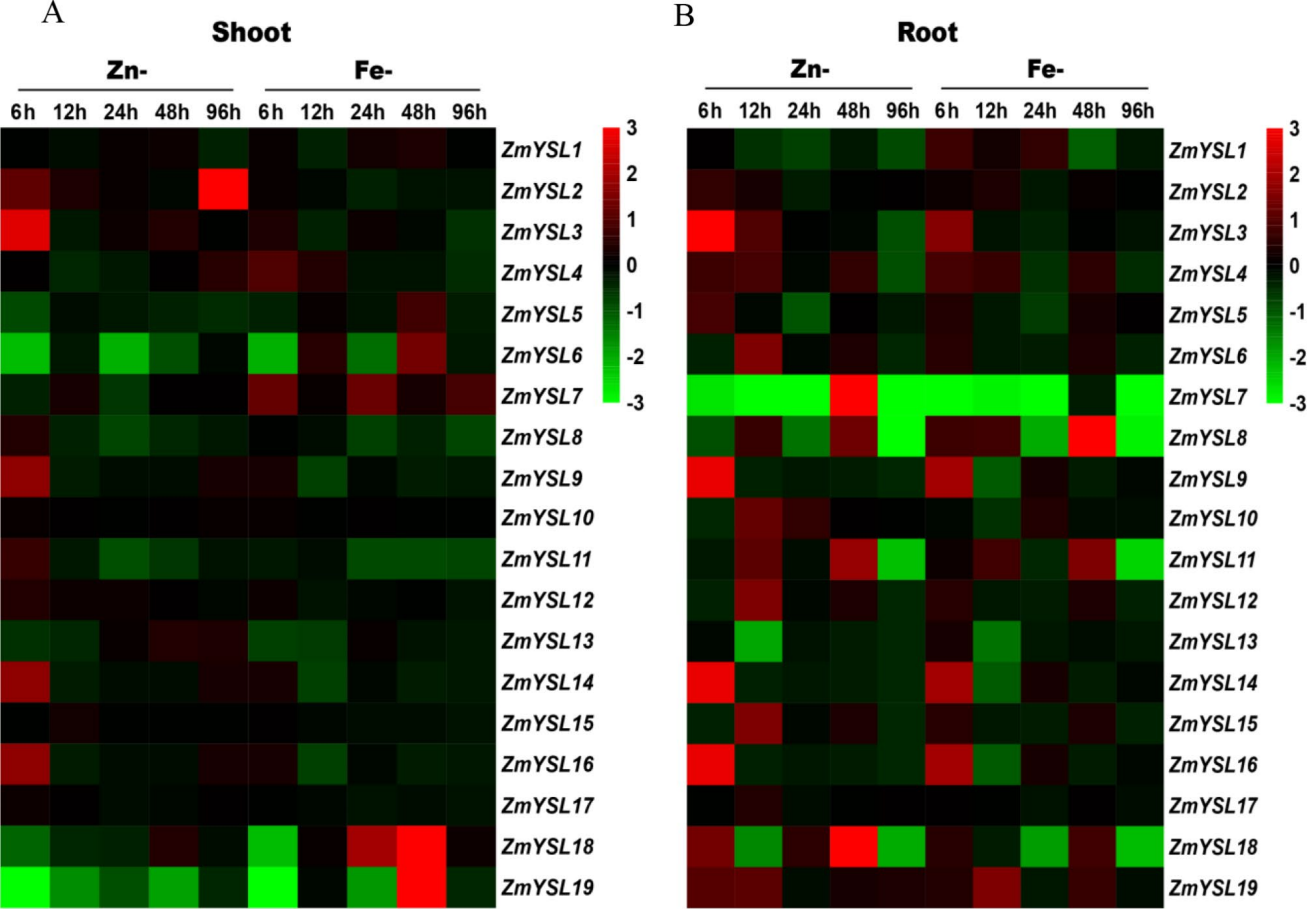
Expression profiles of the *YSL* genes of maize under iron/zinc deficiency. A heatmap of the relative expression patterns of the 19 *ZmYSL* genes in the root and shoot of maize at the three-leaf stage. The seedlings were treated in the Hoagland nutrient solution without iron and zinc for 0, 6, 12, 24, 48, and 96 h. *ZmActin1* was used as the reference gene. The relative expression value was normalized through the gene expression value at 0 h. The color bar on the right of the heatmap indicates the change; green represents inhibition under the deficiency conditions, black represents no change, and red represents induction under deficiency conditions

YSL transporters are involved in the acquisition and subsequent transport of Fe/Zn in leaves. To evaluate the response of the YSL transporters of maize to Fe and Zn deficiency, we examined their expression in the roots and shoots. In the roots, *ZmYS1*, *ZmYSL5*, *ZmYSL8*, *ZmYSL9*, *ZmYSL11*, *ZmYSL16*, and *ZmYSL19* were induced by iron deficiency, while the expression of *ZmYS1* and *ZmYSL8* was suppressed by zinc deficiency (Fig. 8 and Fig. S1). The level of expression of *ZmYSL4* and *ZmYSL11* was induced by iron and zinc deficiency. Among the other six genes, i.e., *ZmYS1*, *ZmYSL5*, *ZmYSL8*, *ZmYSL9*, *ZmYSL11*, and *ZmYSL16*, the expression levels induced by zinc deficiency were higher than those induced by iron deficiency, except for the expression of *ZmYSL19* (Fig. 8 and Fig. S1). Additionally, *ZmYSL7* and *ZmYSL13* were suppressed under zinc and iron deficiency. In the roots, seven genes, including *ZmYSL6*, *ZmYSL10*, *ZmYSL12*, *ZmYSL14*, *ZmYSL15*, *ZmYSL17*, and *ZmYSL18*, were induced under zinc deficiency, but their expression showed negligible changes under iron deficiency (Fig. 8 and Fig. S1).

In the shoot, *ZmYSL5* and *ZmYSL8* were suppressed under zinc and iron deficiency. *ZmYSL10* and *ZmYSL17* were unaffected by zinc or iron deficiency. The level of expression of *ZmYSL14* was higher under zinc deficiency than under iron deficiency (Fig. 8 and Fig. S1). *ZmYS1*, *ZmYSL4*, *ZmYSL7*, and *ZmYSL18* were induced by iron deficiency, whereas *ZmYSL2*, *ZmYSL3*, *ZmYSL9*, *ZmYSL12*, and *ZmYSL16* were induced by zinc deficiency (Fig. 8 and Fig. S1). *ZmYSL6*, *ZmYSL13*, and *ZmYSL19* were induced by iron deficiency, but their expression was suppressed under zinc deficiency, whereas, *ZmYSL11* and *ZmYSL15* showed the opposite pattern (Fig. 8 and Fig. S1). Some genes were either suppressed or remained unchanged under zinc and iron deficiency conditions, which suggested that they might transport divalent metal ions other than zinc and iron.

### Subcellular localization of YSLs in maize

**Fig. 9:**
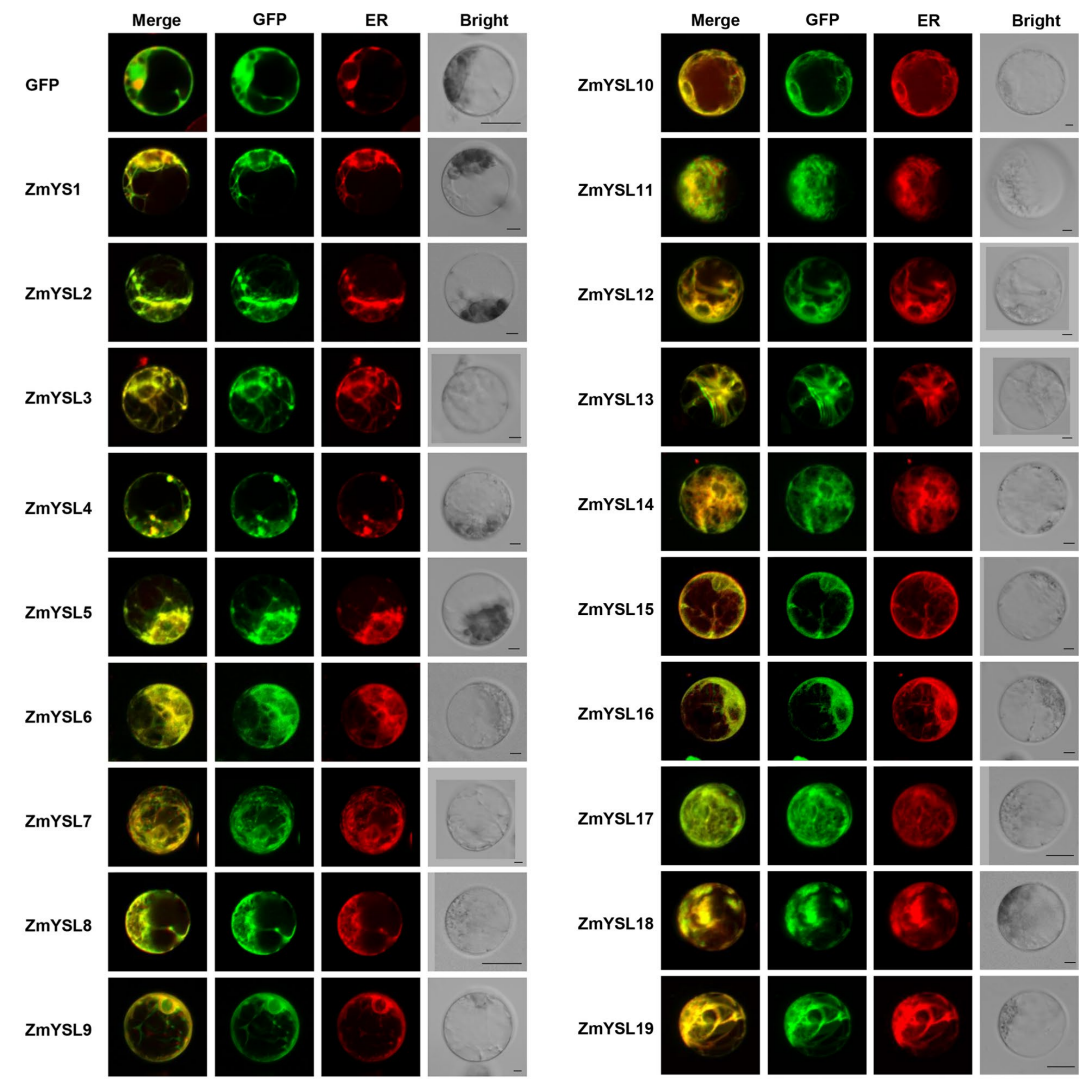
Subcellular localization of 19 ZmYSL::GFP fusion proteins in maize mesophyll protoplasts. GFP was fused with the C-terminal of each YSL, and the fusion proteins were co-expressed with a mCherry-fused ER marker in maize mesophyll protoplasts. Green represents the GFP signal, and red represents the ER marker. The images were obtained using a confocal microscope. The cytoplasmic localization of GFP was used as a control. The scale bar represents 5 μm

Three network forecasting programs, including TargetP (Table S3), Plant-mPLoc (Table S4), and WoLF PSORT (Table S5), predicted that all ZmYSLs would be localized in the plasma membrane. To confirm this prediction, we conducted transient expression assays in maize mesophyll protoplasts and verified the subcellular localization of ZmYSL proteins. The results showed that the ZmYSL-GFP fusion proteins had a ring of fluorescent markers at the plasma membrane site (Fig. S2), and some of the signal was co-localized with the ER-mCherry protein, suggesting that these ZmYSLs were located in the plasma membrane and ER (Fig. 9).

For transmembrane proteins, we also found that the localization signal on the endoplasmic reticulum was probably due to the retention of the unfolded ZmYSL-GFP protein in this compartment. This could be a protective mechanism in cells to prevent too many transmembrane proteins from being anchored and inserted into the plasma membrane.

## Discussion

YSL proteins play a crucial role in the uptake and transport of Fe and Zn, particularly the long-distance transport and mobilization of micronutrients from the soil to the vegetative and reproductive organs of plants. The proteins in the YSL family have been identified in several species, including *Arabidopsis*, barley, and rice, among others. Due to the lack of functional information on YSL proteins in maize, we systematically identified 19 *ZmYSL* genes.

### The first two subgroups of YSL proteins are important for the uptake, transport, and distribution of Zn and Fe

In *Arabidopsis*, AtYSL proteins mainly participate in the transport of metals. The first subgroup participates in the transportation of metals, while the second subgroup controls the excessive accumulation and detoxification of metals. In rice, OsYSL in the first subgroup participates in the uptake, transport, distribution, and accumulation of Zn and Fe [16, 18, 26–29, 41–44]. The second subgroup regulates the excessive accumulation and detoxification of metals. These results suggested that the first and second subgroups can form a relatively complete internal and external cycle of iron homeostasis [21, 23, 30]. In maize, the first subgroup contains ZmYS1, ZmYSL2, and ZmYSL3 proteins (Fig. 3). *ZmYS1* is mainly expressed in the roots and young leaves and is involved in iron uptake and distribution [12]; its function is similar to that of *OsYSL15* in rice [26, 27]. It plays an important role in Fe uptake in roots. The *ys1* mutant exhibits a strong etiolated phenotype, and it can even lead to the death of plants under homozygous conditions [13, 32]. *ZmYSL2* is mainly expressed in the embryo and endosperm and has functions similar to those of OsYSL2 and OsYSL9 in rice [14]. ZmYSL2 plays a key role in the development of maize grain. The *ysl2* (*shrunken4*) mutant lines exhibit severe grain shrinkage; they also show abnormal starch accumulation and mitochondrial structure in the grains [14, 15]. *ZmYSL3* is expressed in all tissues (Fig. 7), especially in aboveground organs, and has functions similar to those of *OsYSL16* in rice. It might be related to the transport and distribution characteristics of aboveground organs. *OsYSL15* and *OsYSL2* are located close to each other on chromosome 4 of rice, while *ZmYS1* and *ZmYSL2* are closely located on chromosome 5 of maize (Fig. 2). Similarities in the distribution of pairings of maize and rice genes on chromosomes are significant for investigating the functions of YSL in the first subgroup of maize. *OsYSL6*, *AtYSL4*, and *AtYSL6* belong to the second subgroup; they prevent excessive metal storage and detoxify excess metals [21, 30]. *ZmYSL4* and *ZmYSL5* belong to the second subgroup and are expressed in various tissues and developmental stages in maize (Figs. 1A and 7). They might control excessive accumulation and detoxification of metals; their functions might be similar to those of the second subgroup proteins in rice. These results indicated that the first and second subgroups may play crucial roles in the homeostasis of metal ions in plants.

### Two highly variable regions of YSL proteins are associated with substrate selectivity and transport

Topological studies showed that all YSL transporters in maize are transmembrane proteins (Fig. 4). ZmYSL transporters possess 11–16 transmembrane domains (Table S6). The first highly variable region of YSL is located between the sixth and seventh transmembrane domains, suggesting a correlation between the helicity of the ring and substrate specificity [32]. Another hypervariable region is the N-terminal of YSL proteins, and the REKELELELER sequences in the N-terminal are similar to the REGLE sequences that are involved in Fe (III) transport [12]. These sequences might be associated with the transport of metal ions. In maize, the N-terminal region included the first highly variable region, and the N-terminal of all 16 YSL proteins had more than 29% glutamate residues (Table 2). ZmYSL2 and ZmYSL11 had the highest number of glutamate residues (50%), and ZmYSL9 contained 32% glutamate. Additionally, ZmYSL3, ZmYSL8-ZmYSL11, ZmYSL13, and ZmYSL17 also showed a significant number of histidine residues. The glutamate and histidine residues found in the N-terminal region may play a role in identifying metal ions. The imidazole group in histidine can form coordination compounds with Fe or other metal ions, thus promoting iron absorption. Additionally, the peptide motif Glu-Xaa-Xaa-Glu was found to play a role in the direct binding of ferric iron in several proteins involved in iron transport, sensing, and storage [45]. The glutamate and histidine residues together form a functional domain for the iron-binding site [46]. The loop ring between the seventh and eighth transmembrane domains is the second highly variable region (Fig. 4). The helicity of this ring might be related to substrate specificity, and it contains more charged residues than other regions. The loop ring might be involved in substrate recognition of trivalent and divalent metal chelate complexes or interactions with other proteins [47].

**Table 2:** Amino acid analysis in hypervariable region

	Glu^1^	His^1^	α-helix^2^	Charged residues^2^
ZmYS1	48%	10%	32%	33%
ZmYSL2	50%	0%	37%	38%
ZmYSL3	33%	31%	36%	37%
ZmYSL4	24%	0%	40%	31%
ZmYSL5	11%	13%	41%	33%
ZmYSL6	43%	0%	22%	26%
ZmYSL7	40%	22%	36%	24%
ZmYSL8	47%	43%	39%	29%
ZmYSL9	32%	43%	25%	25%
ZmYSL10	41%	33%	28%	25%
ZmYSL11	50%	29%	48%	27%
ZmYSL12	35%	14%	25%	33%
ZmYSL13	35%	27%	39%	34%
ZmYSL14	30%	0%	23%	33%
ZmYSL15	29%	0%	40%	35%
ZmYSL16	42%	13%	15%	31%
ZmYSL17	40%	25%	75%	44%
ZmYSL18	31%	0%	36%	33%
ZmYSL19	20%	8%	30%	37%

^1^Glu and His columns mean the ratio of the number of corresponding amino acids in the N-terminal variable region to the number in the total protein

^2^The α-helix and charged residues columns mean the ratio of helix amino acids or the charged residues in the loop between the seventh and eighth transmembrane domain

### Studying the genes of the ZmYSL family provides a theoretical basis and genetic resources for the biofortification of zinc and iron in crops

**Fig. 10:**
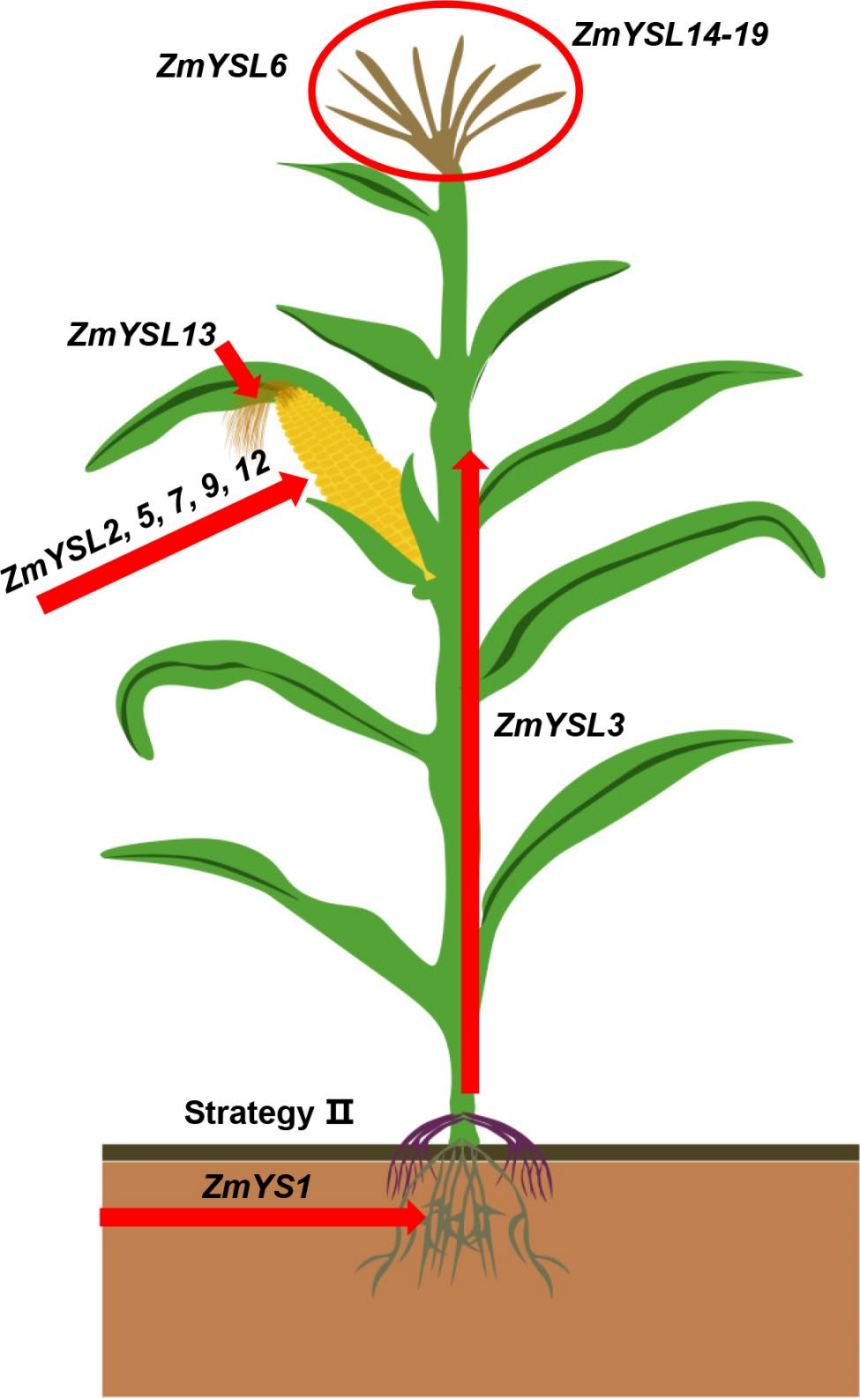
A schematic representation of the expression patterns of ZmYSLs and their specific physiological functions. *ZmYS1* is involved in the uptake of Fe in maize. *ZmYSL3* is involved in Fe transport in maize. *ZmYSL2*, *5*, *7*, *9*, and *12* are involved in Fe and Zn transport during the development of the maize kernel. *ZmYSL13* is involved in Zn transport during silk development in maize. *ZmYSL6* and *ZmYSL14*–*19* are involved in Fe and Zn transport during tassel development in maize

The co-expression of *AtFRD3*, *AtNAS1*, and *PvFER* in rice led to an increase in the iron content by 3.5–5.5 times and an increase in the zinc content by 1.78–2.44 times in polished rice of a transgenic line [48]. Additionally, knocking out *OsVIT2* led to an increase in iron concentration in polished rice [49, 50]. *Cassava* is the primary source of starch for African people. The overexpression of *AtVIT1* in cassava led to an increase in the iron content by 3–7 times in storage roots. The co-expression of *AtIRT1* and *AtFER1* in *Cassava* also resulted in an increase in the iron content by 7–18 times and an increase in the zinc content by 3–10 times [51]. These three examples showed the feasibility and importance of fortifying crops with zinc and iron. Our study provided evidence for the synergistic action of YSL transporters in maize, which are widely distributed across different developmental stages (Fig. 10). Searching for specific transporters responsible for iron reactivation during maize kernel development may be a crucial step toward achieving micronutrient biofortification. In this work, bioinformatics analysis such as phylogenetic tree analysis, chromosome localization and collinearity analysis provided a preliminary understanding of YSL family genes in maize (Figs. 1, 2, 3 and 4). Moreover, the results of collinearity analysis between B73 and Teosinte showed that the four genes (*ZmYSL2*, *ZmYSL6*, *ZmYSL12* and *ZmYSL16)* are highly conserved in evolution, suggesting that these genes play an important role in the transport of zinc/iron in maize. In these four conserved genes, *ZmYSL2* and *ZmYSL12* are mainly expressed in the seeds. The expression analysis showed that *ZmYSL5*, *ZmYSL7*, and *ZmYSL9* are also expressed in seeds, *ZmYS1* is expressed in the roots, *ZmYSL3* is expressed in the roots and the shoots. Co-expression of the above genes in maize by using their native promoters may increase the content of zinc and iron in maize grains. Additionally, combining ZmYSL with other mineral transporters might help in further enhancing the micronutrient content in crops, ultimately improving the yield and quality. It is a convenient strategy for humans to supplement various trace elements through an economical and affordable diet, which can help in solving issues of hunger and hidden hunger.

## Conclusions

In this study, we identified 19 *ZmYSL* genes in maize and determined their phylogenetic relationships, chromosome location, gene structure, protein motif structure, promoter *cis*-regulatory elements, subcellular localization, and gene expression profiles in different tissues and under different levels of iron and zinc. We found that some *ZmYSL* genes are specifically expressed in silk, tassel, and anther, which might be related to the reproductive activity of maize. Some *ZmYSL* genes are expressed in the embryo or endosperm, and they might be related to the development or germination of maize seeds. The expression level of *ZmYSL* genes is regulated by the iron and zinc status, suggesting that they are essential for iron and zinc homeostasis. These results indicated that the *ZmYSL* genes in maize might play a role in biofortification.

## Materials and methods

### Identification of maize ZmYSL family members

We used the known YSL protein sequences of *Arabidopsis*, rice, and ZmYS1 as queries through the tblastn program of NCBI, maizeGDB, and Ensembl Plants to search for all candidate genes. We used the Pfam package to confirm the candidate genes via a transmembrane transport domain (PF03169.15) of ZmYS1.

### Phylogenetic analysis of maize YSLs

The phylogenetic tree of 19 ZmYSL proteins was constructed using MEGA10. To perform multiple sequence alignment, the global alignment program ClustalW was used with the default vacancy penalty score and other parameters. The unrooted phylogenetic tree was constructed using the neighbor-joining (NJ) method. The stability of the tree was tested using the bootstrap method with 1,000 replicates, and the p-distance model was used as the distance model. A phylogenetic tree containing 45 YSL proteins from maize, *Arabidopsis*, and rice was constructed using MEGA10.

### Analysis of gene and protein structure and subcellular localization of YSL proteins

The gene structure was analyzed using the Gene Structure Display Server (GSDS: http://gsds.cbi.pku.edu.cn/). The molecular weight and isoelectric point of the predicted proteins were determined using the *pI*/MW tool on ExPASy. To analyze the subcellular localization of the ZmYSL protein, TargetP, WoLF-PSORT, and Plant-mPLoc were used.

### Chromosome localization and collinearity analysis

We obtained the location of each YSL gene from the NCBI database. Among them, 19 ZmYSL genes of maize were randomly distributed on chromosomes 2, 4, 5, 8, and 10. The chromosome position of each gene was mapped using the MapGene2Chrom tool (version 2.1).

The whole-genome fasta files and the genome annotation .gff3 files of maize, rice, and *Arabidopsis* were downloaded from Ensembl Plants and submitted to one-step MCScanX in the TBtools software package for basic Circos tool to mark the recognized positions on chromosomes.

Recently published database ZEAMAP was used to search syntenic blocks. Set *Zea mays* cultivar B73 chromosomes 2, 4, 5, 8, 10 as given location and choose Teosinte (*Zea mays* ssp. *Mexicana*) for comparison. Blocks were screened based on *ZmYSL* location on the chromosomes then search the collinearity of ZmYSL from the blocks [52].

### Transmembrane domain analysis

Multiple sequence alignment was performed using ClustalX (V2.0), and the output result was formatted using Boxshade. The transmembrane domain and the terminal localization of the YSL proteins were predicted using the TMHMM and TMPred prediction programs.

### Detection of conserved domains and motifs

The NCBI-CDD tool and multiple Em for the Motif Elicitation (MEME) program (v5.3.3) were used to identify putative conservationin the ZmYSL protein motifs. The following parameters were specified for running MEME: maximum motif width = 50, minimum motif width = 6, and maximum number of motifs = 14.

### Analysis of the promoter *cis*-regulatory elements

First, the 2,000 bp sequence upstream of the translation start codon (ATG) of 19 *ZmYSL* genes was extracted from the genome file and genome annotation file by the FASTA extraction tool in TBtools. The 2,000 bp promoter sequences were used as queries and submitted to the PlantCARE database. The predicted and analyzed results of the *cis*-regulatory elements of the promoter were evaluated using TBtools for visualization. Additionally, the FASTA file also submitted to MEME-AME to analyze the motifs enriched at the promoter. The motif database was selected the *Arabidopsis* DAP motifs.

### Plant materials and growth conditions

The maize inbred line B73 was used for this study. The B73 seeds had 19.16 mg/kg Zn and 19.42 mg/kg Fe. The B73 inbred line was planted in the field. For analyzing the expression patterns, we collected the roots, stems, leaves, and sheaths at the flare opening stage, while tassels, anthers, ears, cobs, silks, and husks were collected during the flowering period. Embryos and endosperms were sampled at 10, 15, and 20 DAP (Days After Pollination).

For analyzing the expression patterns under Zn or Fe deficiency, the B73 seeds were rolled in germination paper and incubated in the dark at 28 °C. Once the plumules emerged from the germination paper, they were transferred to a light incubator (16 h of light and 8 h of darkness at 26 °C). After 13 days, the seedlings were transferred to the standard Hoagland nutrient solution, Zn-deficient (ZnSO4) Hoagland medium, and Fe-deficient (Fe(III)-EDTA) Hoagland medium and grown for six days. The shoots and roots of the seedlings were collected after 0, 6, 12, 24, 48, and 96 h of treatment and quickly frozen with liquid nitrogen for further study. The frozen samples were stored at − 80 °C.

### RNA isolation, cDNA preparation, and qRT-PCR analysis

Total RNA was extracted using the Trizol kit (Transgen), and the concentration and quality of the RNA samples were checked using a Nanodrop spectrophotometer. The synthesis of cDNA and qRT-PCR were performed following previously published methods. For the qRT-PCR analysis, gene-specific primers were designed as indicated in Table S7. To normalize the relative abundance of mRNA, *ZmActin1* [GenBank: J01238.1] was used as the internal control. Relative gene expression was calculated using the 2^−ΔΔCT^ method.

### Expression patterns

To validate our findings, we used publicly available RNA-Seq data to construct a heatmap illustrating the relative expression patterns of the 19 ZmYSL genes in different tissues and developmental stages of maize, including roots, leaves, SAM (Shoot Apical Meristems) & stems, reproductive organs, and seeds across 80 different stages. The FPKM (Fragments Per Kilobase of exon model per Million mapped fragments) values were normalized for each gene.

### Cloning of target genes

The cDNA sequences of the ZmYSL genes with complete ORFs were retrieved from the MaizeSequence database (http://www.maizesequence.org/). The ORFs of the ZmYSL genes were amplified using the primers listed in Table S8. The cDNA was synthesized following the manufacturer’s instructions (TransGen Biotech Co., Ltd., China), and the high-fidelity DNA polymerase KOD FX Neo (TOYOBO) was used for amplifying the target genes. PCR amplification was conducted using a DNA amplification machine (Eppendorf). The PCR products were separated on a 1.0% agarose gel and purified with the FastPure Gel DNA Extraction Mini Kit (Vazyme) following the manufacturer’s instructions. The purified product was cloned into the pEASY-Blunt vector (TransGen Biotech Co., Ltd., China) and sequenced (Openlab, China).

### Subcellular localization experiment

Gene-specific primers were designed for ZmYSLs, and the stop codon was removed (Table S8). The coding region of ZmYSLs without the stop codon was cloned into the N-terminus of the pRTL2-nGFP vector to generate pRTL2-ZmYSLGFP. The plasmids pRTL2-GFP and pRTL2-ZmYSLGFP were extracted and purified using the Wizard Plus Minipreps DNA Purification System (Promega). To transform the protoplast of the mesophyll cells of maize, the ZmYSL-GFP fusion plasmid and the mCherry-labeled ER marker were co-transformed following a previously described protocol. The mesophyll cells were incubated in the dark at 26 °C for 16 h and observed under a confocal microscope (LSM700, Carl Zeiss). The GFP and mCherry signals were imaged using an excitation laser at 488 and 555 nm, respectively, and a collection laser at 500–530 and 610 nm.

### Electronic supplementary material

Below is the link to the electronic supplementary material.

Supplementary Material 1

Supplementary Material 2

Supplementary Material 3

Supplementary Material 4

Supplementary Material 5

Supplementary Material 6

Supplementary Material 7

Supplementary Material 8

Supplementary Material 9

Supplementary Material 10

Supplementary Material 11

## Data Availability

The data that support the findings of this study are available from the corresponding author upon reasonable request.

## Abbreviations

DMA: Deoxymugineic acid
DAP: Days after pollination
NA: Nicotianamine
ER: Endoplasmic reticulum
Fe: Iron
Fe^2+^: Ferrous
Fe^3+^: Ferric
FRO: Ferric Reductase
GFP: Green fluorescent protein
HMM: Hidden markov model
IRT1: Iron-regulated transporter 1
MAs: Mugineic acid family phytosiderophores
Nramp: Natural Resistance-Associated Macrophage Protein
OPT: Oligopeptide transporter
PS: Phytosiderophores
qRT-PCR: Quantitative reverse transcription polymerase chain reaction
RNA-seq: RNA sequencing
TOM: MAs efflux transporter
YS1: Yellow Stripe 1
YSL: Yellow Stripe 1-Like
ZIP: Zinc-regulated transporters, Iron-regulated transporter-like Protein
