# Supplementary material for: Identification and characterization of yellow stripe-like genes in maize suggest their roles in the uptake and transport of zinc and iron
Source: BMC Plant Biol. 2024 Jan 2;24:3. doi: 10.1186/s12870-023-04691-0 (PMC10759363; doi:10.1186/s12870-023-04691-0)
Supplement: Supplementary file 1 — Supplementary Material 1 [file 12870_2023_4691_MOESM1_ESM.docx]

**Accession number**

AtYSL1: AT4G24120, AtYSL2: AT5G24380, AtYSL3: AT5G53550, AtYSL4: AT5G41000, AtYSL5: AT3G17650, AtYSL6: AT3G27020, AtYSL7: AT1G65730, AtYSL8: AT1G48370; OsYSL1: Q9FTU1.1, OsYSL2: Q6H3Z6.2, OsYSL3: Q6AVD0.2, OsYSL4: Q688S6.1, OsYSL5: Q7XRV1.3, OsYSL6: Q7XRV2.1, OsYSL7: Q6ZGM7.1, OsYSL8: Q0E4J6.1, OsYSL9: Q7XUJ2.2, OsYSL10: Q0J932.2, OsYSL11: Q7X660.1, OsYSL12: Q5JQD7.2, OsYSL13: Q7XKF4.2, OsYSL14: Q6H7J6.1, OsYSL15: Q6H3Z3.1, OsYSL16: Q7XN54.2, OsYSL17L Q6ZCX1.1, OsYSL18: Q941V3.1；ZmYSL1: Zm00001d026604, ZmYSL2: Zm00001d025889, ZmYSL3: Zm00001d025887, ZmYSL4: Zm00001d025888, ZmYSL5: Zm00001d002797, ZmYSL6: Zm00001d003941, ZmYSL7: Zm00001d003939, ZmYSL8: Zm00001d002974, ZmYSL9: Zm00001d002972. ZmYSL10: Zm00001d051193, ZmYSL11: Zm00001d054041, ZmYSL12: Zm00001d054042, ZmYSL13: Zm00001d017429, ZmYSL14: Zm00001d017427, ZmYSL15: Zm00001d017323, ZmYSL16: Zm00001d012466, ZmYSL17: Zm00001d009025, ZmYSL18: Zm00001d010034, ZmYSL19: Zm00001d010036.
