# Supplementary material for: Identification and characterization of yellow stripe-like genes in maize suggest their roles in the uptake and transport of zinc and iron
Source: BMC Plant Biol. 2024 Jan 2;24:3. doi: 10.1186/s12870-023-04691-0 (PMC10759363; doi:10.1186/s12870-023-04691-0)
Supplement: Supplementary file 2 — Supplementary Material 2 [file 12870_2023_4691_MOESM2_ESM.docx]

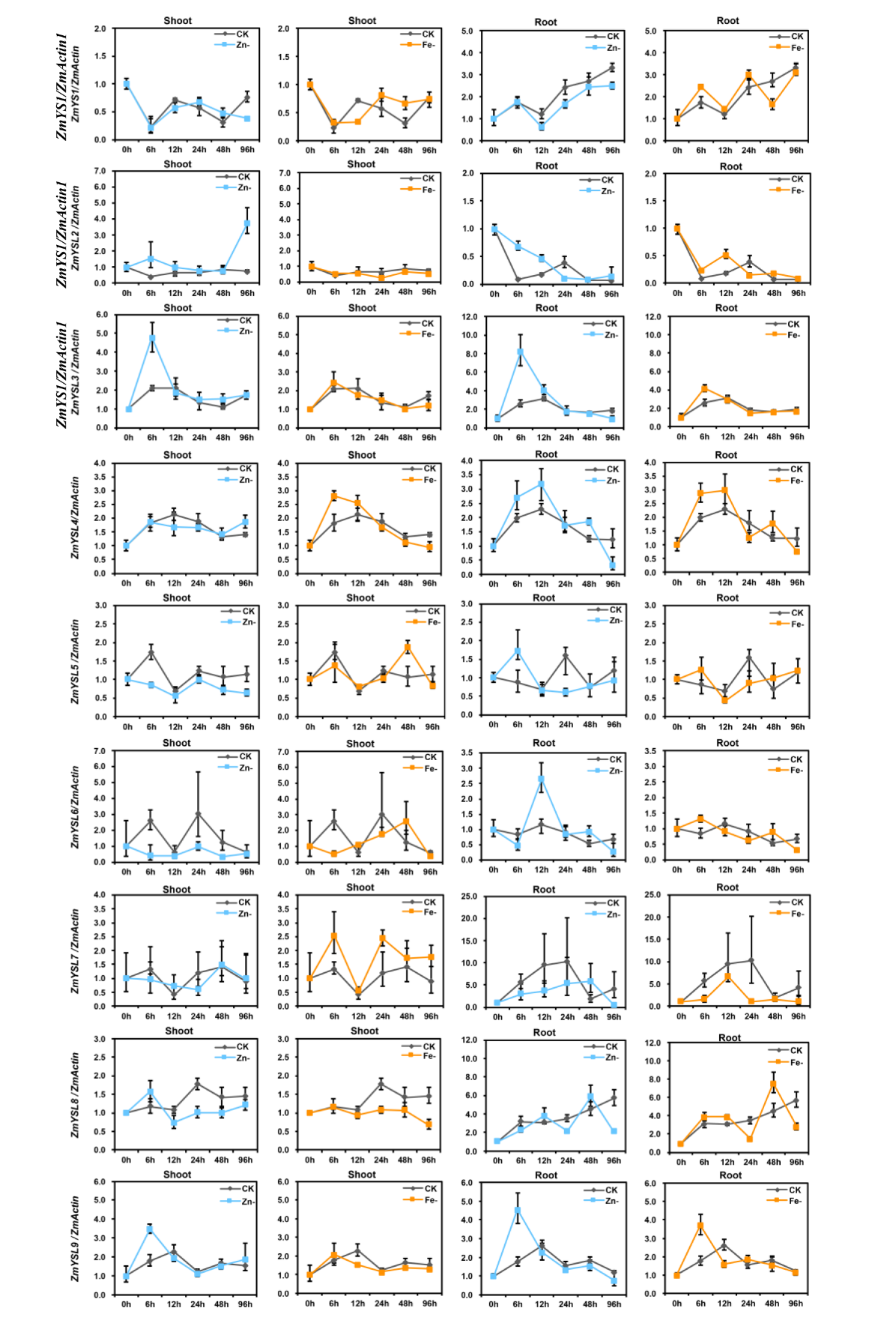


**Fig. S1 Expression profiles of *ZmYSLs* in response to zinc and iron deficiency.** The maize seedlings were cultured to the three-leaf stage in standard Hoagland solution and then transferred to Hoagland solution with Fe deficiency (Fe-) and Zn deficiency (Zn-) treatments, respectively. The shoots (S) and roots (R) were harvested at 0, 6, 12, 24, 48 and 96 h after treatments. *ZmActin1* as the reference gene. The error bars indicate standard deviations.


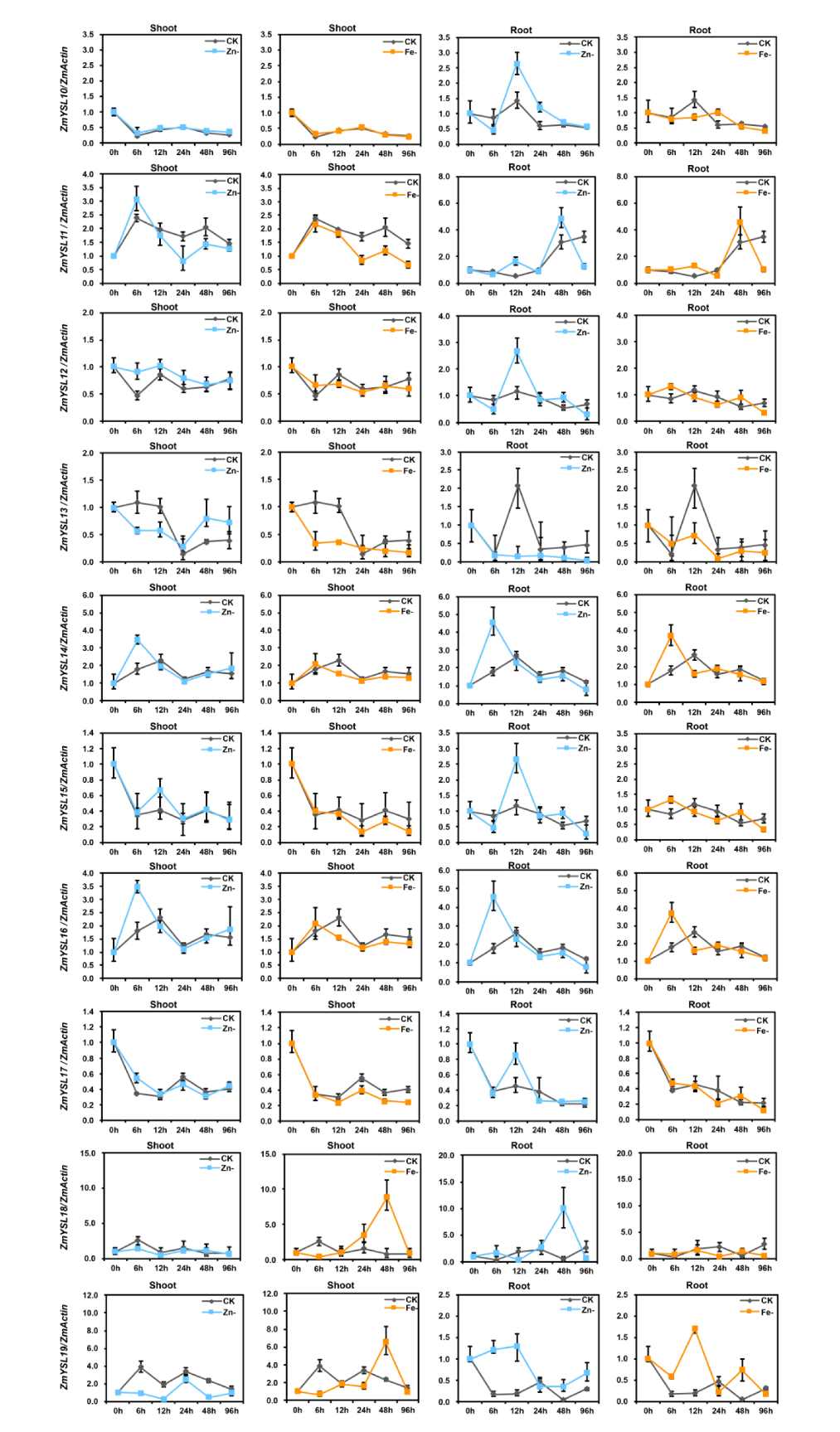


**Fig. S1 (Continue) Expression profiles of *ZmYSLs* in response to zinc and iron deficiency.**
