# Supplementary material for: Identification and characterization of yellow stripe-like genes in maize suggest their roles in the uptake and transport of zinc and iron
Source: BMC Plant Biol. 2024 Jan 2;24:3. doi: 10.1186/s12870-023-04691-0 (PMC10759363; doi:10.1186/s12870-023-04691-0)
Supplement: Supplementary file 3 — Supplementary Material 3 [file 12870_2023_4691_MOESM3_ESM.docx]

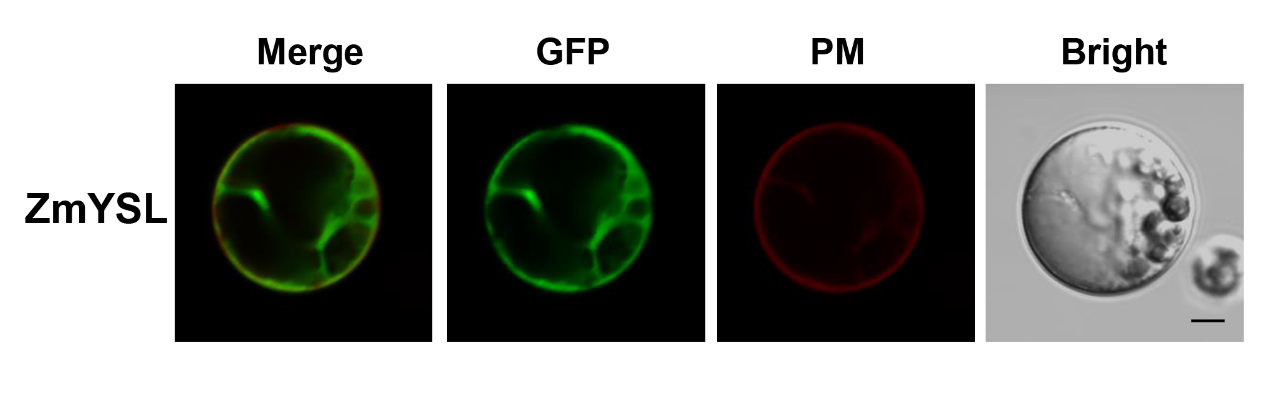
**Fig. S2 Colocalization of ZmYSL::GFP and PM::mCherry in maize mesophyll protoplasts.** GFP was fused with the C-terminal of each YSL and the fusion proteins were co-expressed with a mCherry-fused PM (plasma membrane) marker in maize mesophyll protoplasts. The GFP signal is indicated in green, the ER marker is indicated in red. The images were obtained by a confocal microscope. The scale bar represents 5 μm.
