# Supplementary material for: Identification and characterization of yellow stripe-like genes in maize suggest their roles in the uptake and transport of zinc and iron
Source: BMC Plant Biol. 2024 Jan 2;24:3. doi: 10.1186/s12870-023-04691-0 (PMC10759363; doi:10.1186/s12870-023-04691-0)
Supplement: Supplementary file 4 — Supplementary Material 4 [file 12870_2023_4691_MOESM4_ESM.docx]

Table S1 Distribution of cis-elements detected by PlantCARE on each ZmYSL promoter

| gene | ABA responsiveness element | | | Light response | | | Anoxic induced | | Zein metabolism regulation | meristem expression | |
| --- | --- | --- | --- | --- | --- | --- | --- | --- | --- | --- | --- |
|  | ABRE3a | ABRE4 | DRE1 | G-box | I-box | ATC-motif | ARE | GC-motif | O2-site | OCT | dOCT |
|  | TACGTG | CACGTA | ACCGAGA | CACG(A/T)C | gGATAAGGTG | TGCTATCCG | AAACCA | CCCCCG | GATGA(C/T)(A/G)TG(A/G) | CGCGGATC | CTCGGATC |
| ZmYS1 | 1 |  | 1 |  |  |  | 2 |  |  |  |  |
| ZmYSL2 |  |  |  |  |  |  | 1 |  |  |  |  |
| ZmYSL3 |  |  |  |  |  |  |  | 2 | 1 |  |  |
| ZmYSL4 |  |  |  | 2 |  |  |  |  | 1 |  |  |
| ZmYSL5 |  |  |  |  | 1 |  |  |  | 1 |  |  |
| ZmYSL6 | 4 | 1 |  | 2 |  |  | 1 | 1 |  |  |  |
| ZmYSL7 |  | 1 |  | 1 |  |  | 2 |  |  |  |  |
| ZmYSL8 |  |  |  | 2 |  |  | 2 | 1 |  |  |  |
| ZmYSL9 |  | 2 |  | 1 | 1 |  | 1 | 1 |  |  |  |
| ZmYSL10 |  |  |  | 2 |  |  | 4 |  |  |  |  |
| ZmYSL11 |  |  |  | 1 |  |  | 2 |  |  |  |  |
| ZmYSL12 | 1 |  |  |  |  |  | 1 |  |  |  |  |
| ZmYSL13 |  | 1 |  | 2 |  |  | 1 |  | 1 |  |  |
| ZmYSL14 | 1 |  |  | 1 |  |  | 1 |  | 1 |  |  |
| ZmYSL15 |  |  |  | 2 |  |  | 1 |  | 1 |  |  |
| ZmYSL16 |  |  |  | 1 |  |  | 3 |  |  | 1 |  |
| ZmYSL17 |  |  |  |  |  |  | 3 | 1 |  |  |  |
| ZmYSL18 |  |  |  |  |  | 1 | 3 |  | 1 |  | 1 |
| ZmYSL19 |  |  |  |  | 1 |  | 1 | 1 |  |  |  |

The promoter sequences are 2000bp upstream of each ZmYSL gene.
