# Supplementary material for: Identification and characterization of yellow stripe-like genes in maize suggest their roles in the uptake and transport of zinc and iron
Source: BMC Plant Biol. 2024 Jan 2;24:3. doi: 10.1186/s12870-023-04691-0 (PMC10759363; doi:10.1186/s12870-023-04691-0)
Supplement: Supplementary file 5 — Supplementary Material 5 [file 12870_2023_4691_MOESM5_ESM.docx]

Table S2 Enrichment motifs detected on ZmYSL promoters

| rank | consensus | logo | motif_ID | *p*-value | TP | PWM_min |
| --- | --- | --- | --- | --- | --- | --- |
| 1 | HTTTTTWTTTTTTTTTTTTWWCTTTTTH | 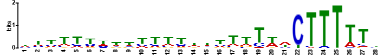 | At4g38000 | 1.03E-09 | 18 | 1.19 |
| 2 | CYTCCTCCTCCTCCTCCTC | 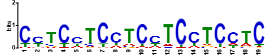 | TF3A | 1.22E-07 | 12 | 1.21 |
| 3 | RARARARAGAGAGAGAGAGAGAGAGAGAG | 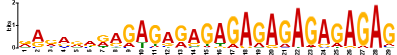 | BPC5 | 5.07E-07 | 4 | 42.3 |
| 4 | WTDWMAAAAAAAAAAAAAAAA | 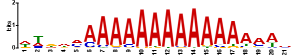 | REM19 | 7.51E-07 | 12 | 1.32 |
| 5 | RAMAAAAAAAAAAAA | 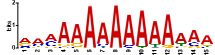 | REM19 | 2.22E-06 | 9 | 10.9 |
| 6 | WHTTTTTTHYTTTTTACTTTTTNHTTTWW | 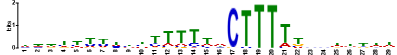 | AT5G66940 | 6.85E-06 | 15 | 1.54 |
| 7 | WCCDCCGCCRCCDCCGCCGCC | 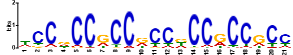 | SHN3 | 1.49E-05 | 7 | 2.28 |
| 8 | TTTWCTTTTTHHYTTTTTTTT | 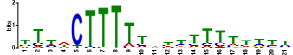 | OBP3 | 2.12E-05 | 10 | 4.33 |
| 9 | YYTTTTDCTTTTTBT | 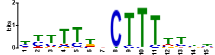 | AT1G47655 | 3.36E-05 | 18 | 1.16 |
| 10 | GDCGGYGGHGRHGRHGGNGR | 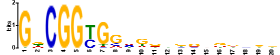 | At4g28140 | 4.37E-05 | 7 | 8.12 |
| 11 | RAAAAAGWAAAAARAAAAARA | 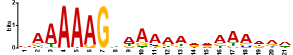 | AT5G02460 | 4.84E-05 | 6 | 25.2 |
| 12 | WCCGCCGCCDYCKCCGCCGCH | 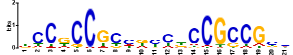 | LOB | 5.53E-05 | 6 | 7.58 |
| 13 | CDCCRCCRCCDCCRCCGYCR | 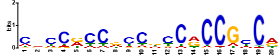 | AT1G71450 | 6.38E-05 | 7 | 5.17 |
| 14 | GGHGGYGGCGGTGGH | 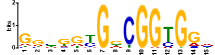 | At1g22810 | 6.76E-05 | 4 | 39.2 |
| 15 | WWTTTTGTCKTTTTSTK | 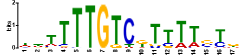 | IDD5 | 9.05E-05 | 11 | 1.71 |
| rank | consensus | logo | motif_ID | *p*-value | TP | PWM_min |
| 16 | VWKTTTTTTTTTTTTTTKB | 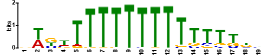 | VRN1 | 1.22E-04 | 8 | 3.35 |
| 17 | YGGCGGHKRYGGCGGCGGMGR | 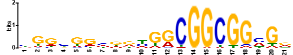 | ERF9 | 1.36E-04 | 4 | 46.3 |
| 18 | RTGRTGRTGRTGRTG | 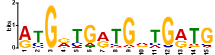 | GATA1 | 1.82E-04 | 10 | 1.71 |
| 19 | WCCACCACCACCWCC | 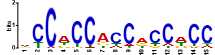 | 3XHMGBOX1 | 2.73E-04 | 7 | 5.17 |
| 20 | GGMGGYGGHGGYGGYGGTGGW | 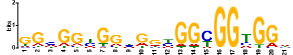 | ERF48 | 3.47E-04 | 6 | 6.19 |
| 21 | DCCKCCGCCGYCDMHDCCKCC | 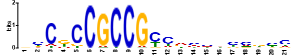 | LEP | 3.71E-04 | 8 | 4.16 |
| 22 | HGGHGGHGGCGGCGGMGGW | 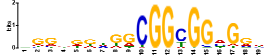 | ESE3 | 3.98E-04 | 5 | 24.4 |
| 23 | WWHTTWTTTTTGTCKTTTTBT | 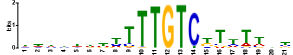 | AtIDD11 | 3.99E-04 | 12 | 1.76 |
| 24 | HHYYTTYTTTTTTWVCTTTTT | 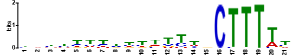 | OBP1 | 5.67E-04 | 11 | 3.33 |
| 25 | TTYTTTTTTTTTWACTTTTTB | 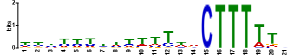 | AT2G28810 | 6.67E-04 | 14 | 1.68 |
| 26 | GMGRHDDWRRCGGCGGMGGHGRNRR | 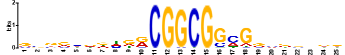 | PUCHI | 8.48E-04 | 5 | 14.7 |
| 27 | YYNYCDCCDCCRCCDCCGCCGCCR | 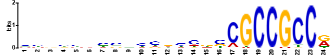 | ERF105 | 8.87E-04 | 8 | 3.34 |
| 28 | CTCTCTCTCTCTCTCTCTCTC | 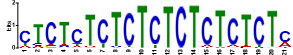 | BPC6 | 1.30E-03 | 2 | 2.33E+04 |
| 29 | GAGAGAGAGAGAGAGAGAGAGAGA | 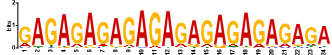 | BPC1 | 1.80E-03 | 3 | 2.23 |
| 30 | CCKCCGCCGCCRCCDCMDCCD | 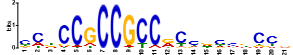 | CRF4 | 2.20E-03 | 4 | 26.5 |
| 31 | BYSACGTCAYCAYC | 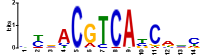 | bZIP50 | 2.21E-03 | 12 | 1.22 |
| rank | consensus | logo | motif_ID | *p*-value | TP | PWM_min |
| 32 | TTTTTTYTTTWNCTTTTTN | 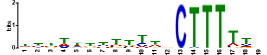 | OBP3 | 2.35E-03 | 3 | 56.3 |
| 33 | CTCTCTCTCTCTCTCTCTCTC | 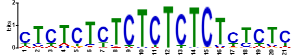 | FRS9 | 2.88E-03 | 3 | 13.6 |
| 34 | YCNCCDYCDYCDCCACCGMC | 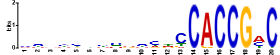 | DREB26 | 3.04E-03 | 5 | 11.5 |
| 35 | RWCGAYGACGW | 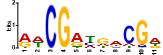 | AT2G15740 | 3.72E-03 | 7 | 2.71 |
| 36 | RCGGCGGHGRWDRNGGNRRNG | 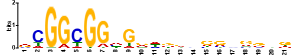 | 3XHMGBOX1 | 4.06E-03 | 11 | 1.56 |
| 37 | NCCDCCNYCDCCGCCGCCRYH | 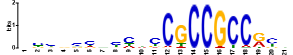 | ERF10 | 4.73E-03 | 9 | 1.05 |
| 38 | WWWWTTTTTGTCGTTTTSTK | 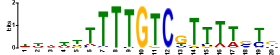 | At1g14580 | 5.59E-03 | 8 | 1.11 |
| 39 | TTTTYACTTTTTYTTTTTTTTTTTTTW | 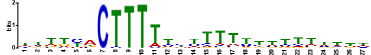 | AT1G69570 | 6.48E-03 | 10 | 2.03 |
| 40 | YCNCCNCCDYCDCCGCCGYCA | 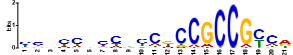 | ERF7 | 6.86E-03 | 9 | 1.29 |
| 41 | MASAAAAMGACAAAAWW | 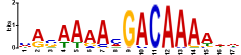 | At1g14580 | 6.97E-03 | 9 | 1.14 |
| 42 | CGSCGGADWWKRCGGCG | 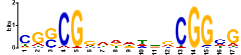 | LBD13 | 7.04E-03 | 8 | 2.52 |
| 43 | YGACGTCABCA | 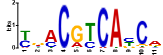 | TGA2 | 7.16E-03 | 12 | 1.16 |
| 44 | WWWWTTTTTGTCKTTTTSTD | 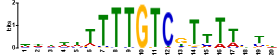 | JKD | 7.40E-03 | 8 | 2.05 |
| 45 | YCDCCDCCDCCGCCGCCRYYD | 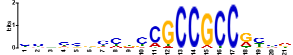 | ERF2 | 8.87E-03 | 8 | 1.13 |
| 46 | CTCTYTCTCTCTCTC | 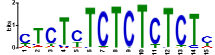 | BPC1 | 9.15E-03 | 4 | 3.81 |
| 47 | CCDCCGCCGCCGCCR | 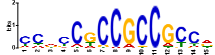 | ERF5 | 9.29E-03 | 5 | 5.06 |
| 48 | CGCCGY | 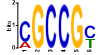 | RAP26 | 9.45E-03 | 3 | 6.01 |
| 49 | CDYCKCCGCCGYCR | 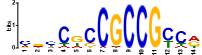 | AT1G28160 | 1.02E-02 | 6 | 3.57 |

Sequence motifs enriched in the promoter regions of ZmYSL genes. The p-value were all less than 0.05. TP means true positive and the number in the TP column indicates the amount of ZmYSL promoters with that motif. The 'PWM score' is computed by scoring the sequence with the motif.
