# Supplementary material for: Identification and characterization of yellow stripe-like genes in maize suggest their roles in the uptake and transport of zinc and iron
Source: BMC Plant Biol. 2024 Jan 2;24:3. doi: 10.1186/s12870-023-04691-0 (PMC10759363; doi:10.1186/s12870-023-04691-0)
Supplement: Supplementary file 6 — Supplementary Material 6 [file 12870_2023_4691_MOESM6_ESM.docx]

Table S3. Prediction results of subcellular localization of TargetP.

| Name | Len | cTP | mTP | SP | other | Loc | RC | TPlen |
| --- | --- | --- | --- | --- | --- | --- | --- | --- |
| ZmYS1 | 682 | 0.018 | 0.253 | 0.011 | 0.973 | _ | 2 | _ |
| ZmYSL2 | 711 | 0.026 | 0.071 | 0.034 | 0.971 | _ | 1 | _ |
| ZmYSL3 | 672 | 0.021 | 0.154 | 0.012 | 0.969 | _ | 1 | _ |
| ZmYSL4 | 668 | 0.179 | 0.042 | 0.074 | 0.876 | _ | 2 | _ |
| ZmYSL5 | 679 | 0.219 | 0.112 | 0.007 | 0.722 | _ | 3 | _ |
| ZmYSL6 | 695 | 0.013 | 0.076 | 0.015 | 0.984 | _ | 1 | _ |
| ZmYSL7 | 738 | 0.102 | 0.065 | 0.371 | 0.099 | S | 4 | 31 |
| ZmYSL8 | 718 | 0.221 | 0.115 | 0.117 | 0.823 | _ | 2 | _ |
| ZmYSL9 | 716 | 0.209 | 0.048 | 0.030 | 0.879 | _ | 2 | _ |
| ZmYSL10 | 721 | 0.215 | 0.057 | 0.097 | 0.860 | _ | 2 | _ |
| ZmYSL11 | 725 | 0.315 | 0.043 | 0.160 | 0.838 | _ | 3 | _ |
| ZmYSL12 | 714 | 0.220 | 0.105 | 0.128 | 0.616 | _ | 4 | _ |
| ZmYSL13 | 727 | 0.143 | 0.091 | 0.290 | 0.743 | _ | 3 | _ |
| ZmYSL14 | 684 | 0.096 | 0.031 | 0.030 | 0.937 | _ | 1 | _ |
| ZmYSL15 | 698 | 0.059 | 0.067 | 0.025 | 0.922 | _ | 1 | _ |
| ZmYSL16 | 707 | 0.138 | 0.130 | 0.171 | 0.694 | _ | 3 | _ |
| ZmYSL17 | 697 | 0.039 | 0.072 | 0.002 | 0.966 | _ | 1 | _ |
| ZmYSL18 | 683 | 0.028 | 0.048 | 0.036 | 0.975 | _ | 1 | _ |
| ZmYSL19 | 679 | 0.024 | 0.056 | 0.032 | 0.983 | _ | 1 | _ |
| cutoff |  | 0.620 | 0.760 | 0.000 | 0.530 |  |  |  |

cope, chloroplast target protein. mTP, mitochondria target protein. SP, secretory protein. The RC value means confidence level varying from 1 to 5. And the larger the value, the less confidence.
