# Supplementary material for: Identification and characterization of yellow stripe-like genes in maize suggest their roles in the uptake and transport of zinc and iron
Source: BMC Plant Biol. 2024 Jan 2;24:3. doi: 10.1186/s12870-023-04691-0 (PMC10759363; doi:10.1186/s12870-023-04691-0)
Supplement: Supplementary file 7 — Supplementary Material 7 [file 12870_2023_4691_MOESM7_ESM.docx]

Table S4. Prediction results of subcellular localization of Plant-mPLoc.

| **Protein ID** | **Score** | **Expected Accuracy** | **Localization Class** | **Gene Ontology Terms*** | **Annotation Type** |
| --- | --- | --- | --- | --- | --- |
| ZmYS1 | 53 | 91% | plasma membrane | integral to membrane; plasma membrane; | PSI-BLAST |
| ZmYSL2 | 52 | 90% | plasma membrane | integral to membrane; plasma membrane; | PSI-BLAST |
| ZmYSL3 | 61 | 93% | plasma membrane | integral to membrane; plasma membrane; | PSI-BLAST |
| ZmYSL4 | 41 | 89% | plasma membrane | integral to membrane; plasma membrane; | PSI-BLAST |
| ZmYSL5 | 36 | 87% | plasma membrane | integral to membrane; plasma membrane; | PSI-BLAST |
| ZmYSL6 | 42 | 89% | plasma membrane | integral to membrane; plasma membrane; | PSI-BLAST |
| ZmYSL7 | 42 | 89% | plasma membrane | integral to membrane; plasma membrane; | PSI-BLAST |
| ZmYSL8 | 46 | 89% | plasma membrane | integral to membrane; plasma membrane; | PSI-BLAST |
| ZmYSL9 | 46 | 89% | plasma membrane | integral to membrane; plasma membrane; | PSI-BLAST |
| ZmYSL9 | 46 | 89% | plasma membrane | integral to membrane; plasma membrane; | PSI-BLAST |
| ZmYSL10 | 46 | 89% | plasma membrane | integral to membrane; plasma membrane; | PSI-BLAST |
| ZmYSL11 | 45 | 89% | plasma membrane | integral to membrane; plasma membrane; | PSI-BLAST |
| ZmYSL12 | 44 | 89% | plasma membrane | integral to membrane; plasma membrane; | PSI-BLAST |
| ZmYSL13 | 43 | 89% | plasma membrane | integral to membrane; plasma membrane; | PSI-BLAST |
| ZmYSL14 | 36 | 87% | plasma membrane | integral to membrane; plasma membrane; | PSI-BLAST |
| ZmYSL15 | 32 | 86% | plasma membrane | integral to membrane; plasma membrane; | PSI-BLAST |
| ZmYSL16 | 26 | 84% | plasma membrane | integral to membrane; plasma membrane; | PSI-BLAST |
| ZmYSL17 | 32 | 86% | plasma membrane | integral to membrane; plasma membrane; | PSI-BLAST |
| ZmYSL18 | 22 | 84% | plasma membrane | integral to membrane; plasma membrane; | PSI-BLAST |
| ZmYSL19 | 22 | 84% | plasma membrane | integral to membrane; plasma membrane; | PSI-BLAST |

* Gene Ontology Terms, integral to membrane GO:0016021(IEA); plasma membrane GO:0005886(IDA)
