# Supplementary material for: Identification and characterization of yellow stripe-like genes in maize suggest their roles in the uptake and transport of zinc and iron
Source: BMC Plant Biol. 2024 Jan 2;24:3. doi: 10.1186/s12870-023-04691-0 (PMC10759363; doi:10.1186/s12870-023-04691-0)
Supplement: Supplementary file 8 — Supplementary Material 8 [file 12870_2023_4691_MOESM8_ESM.docx]

Table S5. Prediction results of subcellular localization of WoLF PSORT.

| Gene Name | plas | ER | golg | vacu | nucl | cyto | chlo | totol |
| --- | --- | --- | --- | --- | --- | --- | --- | --- |
| ZmYS1 | 10 | 2 | 2 |  |  |  |  | 14 |
| ZmYSL2 | 10 | 2 |  |  | 1 |  | 1 | 14 |
| ZmYSL3 | 11 |  | 2 |  |  | 1 |  | 14 |
| ZmYSL4 | 10 | 2 |  | 2 |  |  |  | 14 |
| ZmYSL5 | 11 | 1 |  | 1 | 1 |  |  | 14 |
| ZmYSL6 | 11 | 3 |  |  |  |  |  | 14 |
| ZmYSL7 | 11 | 2 |  | 1 |  |  |  | 14 |
| ZmYSL8 | 14 |  |  |  |  |  |  | 14 |
| ZmYSL9 | 10 |  | 2 | 2 |  |  |  | 14 |
| ZmYSL10 | 10 | 1 | 2 | 1 |  |  |  | 14 |
| ZmYSL11 | 13 | 1 |  |  |  |  |  | 14 |
| ZmYSL12 | 11 | 2 |  | 1 |  |  |  | 14 |
| ZmYSL13 | 10 | 1 | 2 | 1 |  |  |  | 14 |
| ZmYSL14 | 12 | 1 |  | 1 |  |  |  | 14 |
| ZmYSL15 | 12 | 1 |  | 1 |  |  |  | 14 |
| ZmYSL16 | 11 | 2 |  | 1 |  |  |  | 14 |
| ZmYSL17 | 9 | 1 | 1 | 3 |  |  |  | 14 |
| ZmYSL18 | 10 | 2 |  | 2 |  |  |  | 14 |
| ZmYSL19 | 13 |  |  | 1 |  |  |  | 14 |

Plas represents the plasma membrane, ER represents the endoplasmic reticulum. Golg represents Golgi apparatus. Vacu represents vacuole. Nulc represents cell nucleus. Cyto represents cytoplasm. Chlo represents chloroplast. The higher score, the higher confidence, giving a total of 14 points.
