# Supplementary material for: Identification and characterization of yellow stripe-like genes in maize suggest their roles in the uptake and transport of zinc and iron
Source: BMC Plant Biol. 2024 Jan 2;24:3. doi: 10.1186/s12870-023-04691-0 (PMC10759363; doi:10.1186/s12870-023-04691-0)
Supplement: Supplementary file 9 — Supplementary Material 9 [file 12870_2023_4691_MOESM9_ESM.docx]

Table S6. Prediction results of transmembrane domains of TMHMM.

| **Gene name** | **length** | **TMAA** | **TMHs** | **N-in** | | **N-term signal** | |
| --- | --- | --- | --- | --- | --- | --- | --- |
| ZmYS1 | 682 | 312 | 14 | 0.97 | in | 7.55 |  |
| ZmYSL2 | 711 | 313 | 14 | 0.97 | in | 0.00 |  |
| ZmYSL3 | 672 | 310 | 12 | 0.99 | in | 0.00 |  |
| ZmYSL4 | 668 | 283 | 13 | 0.18 | out | 11.31 | signal |
| ZmYSL5 | 679 | 327 | 16 | 0.52 | out | 19.11 | signal |
| ZmYSL6 | 695 | 295 | 13 | 0.99 | in | 19.26 | signal |
| ZmYSL7 | 738 | 300 | 13 | 0.86 | in | 0.00 |  |
| ZmYSL8 | 718 | 293 | 12 | 0.98 | in | 0.00 |  |
| ZmYSL9 | 716 | 283 | 12 | 0.96 | in | 0.00 |  |
| ZmYSL10 | 721 | 295 | 12 | 0.79 | in | 0.00 |  |
| ZmYSL11 | 725 | 311 | 14 | 0.96 | in | 0.00 |  |
| ZmYSL12 | 714 | 293 | 12 | 0.90 | in | 0.03 |  |
| ZmYSL13 | 727 | 270 | 11 | 0.52 | out | 0.00 |  |
| ZmYSL14 | 684 | 281 | 12 | 0.67 | in | 21.74 | signal |
| ZmYSL15 | 698 | 315 | 14 | 0.87 | in | 5.90 |  |
| ZmYSL16 | 707 | 301 | 13 | 0.75 | out | 18.65 | signal |
| ZmYSL17 | 697 | 305 | 12 | 0.11 | out | 3.38 |  |
| ZmYSL18 | 683 | 289 | 12 | 0.41 | in | 24.70 | signal |
| ZmYSL19 | 679 | 317 | 13 | 0.40 | out | 26.14 | signal |

TMAA is the expected number of amino acids in a transmembrane helix. N-in column represents the position of n-terminal of the transmembrane helical.
