# Supplementary material for: Identification and characterization of yellow stripe-like genes in maize suggest their roles in the uptake and transport of zinc and iron
Source: BMC Plant Biol. 2024 Jan 2;24:3. doi: 10.1186/s12870-023-04691-0 (PMC10759363; doi:10.1186/s12870-023-04691-0)
Supplement: Supplementary file 10 — Supplementary Material 10 [file 12870_2023_4691_MOESM10_ESM.docx]

Table S7. Primers using in real-time fluorescence quantitative PCR.

| **Primer Name** | **Sequence (5' to 3')** |
| --- | --- |
| YS1-RTF | GCTCTCCGCTGGGTTCTT |
| YS1-RTR | AGGCTCCCGACGCACATA |
| YSL2-RTF | TCAAGCCACCCATCTGCCT |
| YSL2-RTR | GCTCTCATCGCCTGTCGGT |
| YSL3-RTF | TCATTGACAGAAGTAAAGCA |
| YSL3-RTR | CTTCTCCGATTGGACATGGA |
| YSL4-RTF | AAGGAGTGTGATGACTTCGC |
| YSL4-RTR | ATACACAATTAGGTCCTATT |
| YSL5-RTF | ACCGGGAGCAGGCTGATGGT |
| YSL5-RTR | TTCATCAATGGCGCAAACAT |
| YSL6-RTF | TGAAGTTCCTGTCCCGCA |
| YSL6-RTR | CACTGTGCTGTGTGTTGCTT |
| YSL7-RTF | CGCTGCTCTCTCTTTTCAACG |
| YSL7-RTR | GCAATTCAATTTTCTTTCCACA |
| YSL8-RTF | ATGAAGTTCTTGTCCAGGTC |
| YSL8-RTR | ATTTAGCAATCCCTACGACT |
| YSL9-RTF | GCGATCCTCTTCGTGTGGGA |
| YSL9-RTR | TTGCACCAAT AATTACAGGA |
| YSL10-RTF | CGCAGTCCATTCTGGCTCT |
| YSL10-RTR | AGAGCGTGTGCCCACTTGT |
| YSL11-RTF | ACAGAGCCAAAGCAGACACG |
| YSL11-RTR | TTACACTTCCATTTCCACCG |
| YSL12-RTF | CCCCAGGCTGTTCTTTCTCT |
| YSL12-RTR | CCATTGGCACCAGCCTAAAC |
| YSL13-RTF | GCACTACCCGAGGCTATTCT |
| YSL13-RTR | TTCTACTATCAGACTGGCACTCA |
| YSL14-RTF | TCGGCTCGTTCTTCACCATC |
| YSL14-RTR | GGAGAAGACCCTGAGGCACA |
| YSL15-RTF | ACAAGATGCTGGACGACTACA |
| YSL15-RTR | TCCATAAAACTATCTCTTCTTGC |
| YSL16-RTF | TGTGGGGACGGGCTAGGGAT |
| YSL16-RTR | ACAACCCACAACTACGGAAT |
| YSL17-RTF | AGACTGAGCAGATGCGGAAG |
| YSL17-RTR | AAGGAGGAGTGTGTGGGTGT |
| YSL18-RTF | TGGGTTTTGCCGTCATCAGT |
| YSL18-RTR | AGCAGAGCCGTATCCCAACA |
| YSL19-RTF | CAGGCTTAGTATGTGGAGACGG |
| YSL19-RTR | AACGCATCTGCCATCTCCG |
