# Supplementary material for: Identification and characterization of yellow stripe-like genes in maize suggest their roles in the uptake and transport of zinc and iron
Source: BMC Plant Biol. 2024 Jan 2;24:3. doi: 10.1186/s12870-023-04691-0 (PMC10759363; doi:10.1186/s12870-023-04691-0)
Supplement: Supplementary file 11 — Supplementary Material 11 [file 12870_2023_4691_MOESM11_ESM.docx]

Table S8. Primers using in subcellular localization.

| **Primer Name** | **Sequence (5' to 3')** |
| --- | --- |
| XhoI -YS1F | CTCGAGATGGACCTTGCACGGAGCGG |
| XbaI-YS1R | TCTAGAGCTTCCAGGAGTGAACT |
| XhoI-YSL2F | CTCGAGATGGAGATCAACCCCGC |
| XbaI-YSL2R | TCTAGACTAGCTTCCAGGTGTAAATTTCAG |
| XhoI-YSL3F | CTCGAGATGGCGCACGCGCGGGGCGG |
| XbaI-YSL3R | TCTAGAGTGTGAAGACCTGAATG |
| EcoRI-YSL4F | GAATTCATGGGATCGGAGGGTGAGAT |
| XbaI-YSL4R | TCTAGAGCTGCTAAGGGATGGCT |
| XhoI-YSL5F | CTCGAGATGACGCCCGCGGAGACCTC |
| XbaI-YSL5R | TCTAGACCTGGGAGCAGAGGAAG |
| EcoRI-YSL6F | GAATTCATGTCGAGGAGGAGAGCGCA |
| XbaI-YSL6R | TCTAGAGCTGCGGAGGAAAGCGT |
| XhoI-YSL7F | CTCGAGATGAAGCTCAGCCTCACCACGGGCAT |
| XbaI-YSL7R | TCTAGAGGACAAAGTAGCAATGAAATCATCC |
| XhoI-YSL8F | CTCGAGATGGCGCCGCATACCACCGC |
| XbaI-YSL8R | TCTAGAAACTTCCGAGGAAGCCGT |
| XhoI-YSL9F | CTCGAGATGGCGCCGCACACCACCGCTG |
| XbaI-YSL9R | TCTAGATGACAAGCCGAGGAAGCTG |
| XhoI-YSL10F | CTCGAGACGAGCGTCAAACCAAA |
| XbaI-YSL10R | TCTAGACCAATAATTACAGGATGAGAGC |
| XhoI-YSL11F | CTCGAGATGGCCACTGCAACGCACCA |
| XbaI-YSL11R | TCTAGACGATCCAAGAAAAACAT |
| XhoI-YSL12F | CTCGAGCACCATGGCGACTGGCAC |
| XbaI-YSL12R | TCTAGACTAGTTCCCAAGGAAGGTGTCCAC |
| XhoI-YSL13F | CTCGAGATGGCTCCAGCAGCAGGCACCGCCAC |
| XbaI-YSL13R | TCTAGAATTCCCAAGGAAGGAGTCCACTCTGG |
| XhoI-YSL14F | CTCGAGATGGAGGGGTCAGAAGCGGC |
| XbaI-YSL14R | TCTAGATGTGGCGGCTGGGTTTC |
| XhoI-YSL15F | CTCGAGATGGAGTCGGTCGGCGACCC |
| XbaI-YSL15R | TCTAGATCGGGATGCTGCGACTT |
| EcoRI-YSL16F | GAATTCATGAATACAACACATGCAAT |
| XbaI-YSL16R | TCTAGATGTCACAGGCAGTGTAG |
| EcoRI-YSL17F | GAATTCATGGGCGGCGGCGAGCATGA |
| XbaI-YSL17R | TCTAGAACTTGTTCTTCGGCGTG |
| XhoI-YSL18F | CTCGAGATGGACGCCACGATCGGAGAGTCG |
| XbaI-YSL18R | TCTAGATGTCCCCAATGTACTTAAGAACGCATC |
| XhoI-YSL19F | CTCGAGATGGATTCTACAACGGAAGAGGCCTC |
| XbaI-YSL19R | TCTAGATGTCCTTCCATGTGGTCCTAAAGTAT |
